# Application and Evaluation of Interactive 3D PDF for Presenting and Sharing Planning Results for Liver Surgery in Clinical Routine

**DOI:** 10.1371/journal.pone.0115697

**Published:** 2014-12-31

**Authors:** Axel Newe, Linda Becker, Andrea Schenk

**Affiliations:** 1 Chair of Medical Informatics, Friedrich-Alexander-University Erlangen-Nuremberg, Erlangen, Germany; 2 Institute of Psychology, Friedrich-Alexander-University Erlangen-Nuremberg, Erlangen, Germany; 3 Fraunhofer Institute for Medical Image Computing MEVIS, Bremen, Germany; Stanford University Medical Center, United States of America

## Abstract

**Background & Objectives:**

The Portable Document Format (PDF) is the de-facto standard for the exchange of electronic documents. It is platform-independent, suitable for the exchange of medical data, and allows for the embedding of three-dimensional (3D) surface mesh models. In this article, we present the first clinical routine application of interactive 3D surface mesh models which have been integrated into PDF files for the presentation and the exchange of Computer Assisted Surgery Planning (CASP) results in liver surgery. We aimed to prove the feasibility of applying 3D PDF in medical reporting and investigated the user experience with this new technology.

**Methods:**

We developed an interactive 3D PDF report document format and implemented a software tool to create these reports automatically. After more than 1000 liver CASP cases that have been reported in clinical routine using our 3D PDF report, an international user survey was carried out online to evaluate the user experience.

**Results:**

Our solution enables the user to interactively explore the anatomical configuration and to have different analyses and various resection proposals displayed within a 3D PDF document covering only a single page that acts more like a software application than like a typical PDF file (“PDF App”). The new 3D PDF report offers many advantages over the previous solutions. According to the results of the online survey, the users have assessed the pragmatic quality (functionality, usability, perspicuity, efficiency) as well as the hedonic quality (attractiveness, novelty) very positively.

**Conclusion:**

The usage of 3D PDF for reporting and sharing CASP results is feasible and well accepted by the target audience. Using interactive PDF with embedded 3D models is an enabler for presenting and exchanging complex medical information in an easy and platform-independent way. Medical staff as well as patients can benefit from the possibilities provided by 3D PDF. Our results open the door for a wider use of this new technology, since the basic idea can and should be applied for many medical disciplines and use cases.

## Introduction

The human body is a complex organism with a high individual variability, which complicates the exchange of information about the respective peculiarity of a particular patient. One example is the entanglement of the intrahepatic vessel systems and their spatial relationships to pathological structures (e.g. tumors). Preoperative knowledge of the patient-individual anatomy of these structures is a key factor for successful liver interventions [1], [2]. This knowledge can be obtained by the analysis of patient image data and the determination of anatomically precise individual models of the hepatic vessels and their perfusing and draining territories. The creation of these models is the domain of computer assisted surgical planning (CASP) [1], [2]. Results of a CASP need to be communicated with their users, which comprises (A) presenting (visualizing) and (B) sharing the result data itself and the associated auxiliary (or meta) data.

**Figure 1:**
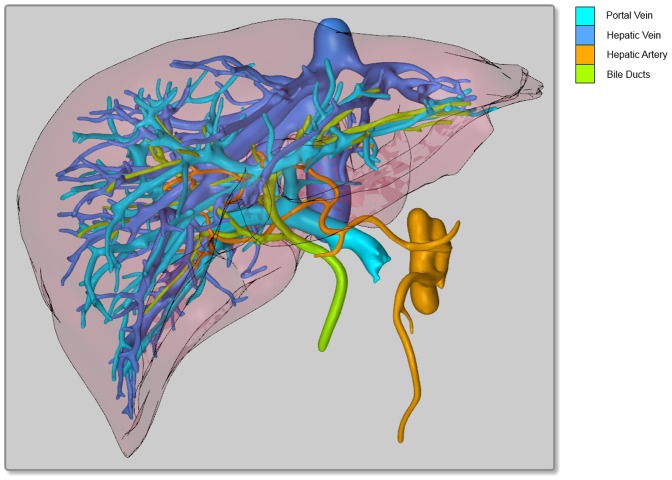
2.5D visualization of the hepatic vessel systems. Portal vein, hepatic vein, hepatic artery & bile ducts are rendered. Even though the vessels are color coded and the trunks are partially cut off, the whole image is still confusing in this projection.

Since a major issue in liver surgery is the three-dimensional (3D) complexity of the intrahepatic vessels and structures, the visualization should be 3D as well [3]. This also applies for other medical applications and leads to a problem: almost all visualization media types that are ubiquitously available nowadays (paper printouts, computer or television screens) only provide a two-dimensional (2D) interface. The common solution is to project the 3D data onto the available 2D plane under acquiescence of the fact that objects can occlude each other in this “2.5D” visualization (Fig. 1) [4]. Although pseudo-3D solutions like 3D television or head-mounted displays can give an impression of 3D and therefore improve the perception of spatial relationships, they do not eliminate the occlusion problem. Besides that, they have some restrictions to take effect (need for 3D glasses, limited viewing angles). True 3D solutions like volumetric displays are still subject of research and not available for the mass market today [5], [6].

The best way to mitigate the occlusion problem is interaction: if the user can select and change the angle of projection, he or she is able to find the best view on the data for the individual objective [4]. Furthermore, interaction also facilitates to perceive and to differentiate spatial relationships of three-dimensional objects much better than by textual description or static 2D renderings [7] if no (pseudo-)3D medium is available.

As long as CASP results (or similar information) are only needed at a single place (e.g. the workstation the CASP was performed on), exchange of the data is not an issue – but in many cases this is not a realistic scenario. While paper printouts or secondary workstations as described in [8] may work within a single hospital, these makeshifts are less than ideal (or fail totally) as soon as the data shall be exchanged with external stakeholders.

Our goal was to create a user-friendly report document that can easily be exchanged and that allows for an interactive exploration of anatomical 3D models without the requirement to install dedicated software or hardware. A quick, efficient, needs-based and easy-to-use access to the planning data was another main focus. Therefore, for our exemplary use case of reporting liver CASP results, we developed a Portable Document Format (PDF) report with embedded high-quality 3D models. This report format was rolled out for use in clinical routine and the user experience was evaluated by means of an online survey.

While the solution presented below focuses on the specific example of liver CASP to proof its practical value and to show the wide range of possible features, we will identify further areas of application and present a broader outlook in the Discussion section.

## Background and Related Work

### The Portable Document Format with Embedded 3D Models

The widely known Portable Document Format is a document description standard (ISO 32000-1:2008, [9]) for definition and reliable reproduction of electronic documents independently of the creating, displaying and printing hardware and software (including operating systems). A PDF file completely describes the content and layout of an electronic document and can encapsulate all necessary resources including texts, fonts, images, multimedia elements and three-dimensional mesh models.

These embedded 3D models – a not particularly well known standard feature of PDF – were proven suitable and useful for electronic publication by several authors. In 2008, the first application in the biomedical sciences was published, showing segmentations of developmental stages of two species obtained from histological images as 3D figures [7]. The first medical application followed in 2011, presenting dental molds, including the (optionally visible) impression body [10]. Using 3D PDF for exchanging anatomy datasets was demonstrated for mouse hearts [11], a human face [12] and the whole human body, comprising of 642 single models [13]. In [14], another proof of concept showed how radiology data in DICOM (Digital Imaging and Communications in Medicine) format can be converted to surface models and then be embedded into PDF. Finally, the feasibility of simulating volume rendering in PDF documents has been demonstrated using the example of a magnetic resonance angiography image [15].

The Adobe Reader (http://get.adobe.com/reader/otherversions/) which is available free of charge for all major operating systems (MS Windows, Mac OS, Linux) offers by default many options to render embedded mesh models and to let the user interact with them (zooming, panning, rotating, selection of components) while displaying the respective PDF document.

Interaction within PDF documents is not limited to 3D models. So called “Interactive Forms” [16], allow for the inclusion of elements (“fields” in terms of the PDF specification) for gathering information interactively from the user. These fields can not only be text input boxes or signature fields, but also other Graphical User Interface (GUI) elements like push buttons, list boxes and combo boxes. Any user interaction with these GUI elements can be linked with the execution of JavaScript code [16], which requires PDF reading software with a built-in JavaScript engine. The Adobe Reader is currently the only free multi-platform PDF reading software that fully supports this and all the other PDF features listed above off-the-shelf and without the need of any extensions.

### Using the Portable Document Format for Exchange of Medical Data

In 2008, the Association for Information and Image Management (AIIM) and the American Society for Testing and Materials (ASTM) released their standard AIIM/ASTM BP-01-2008 “Portable Document Format-Healthcare (PDF) A Best Practices Guide” [17] (also known as PDF Healthcare or PDF/H) which was officially accepted by Adobe [18]. It describes how to use the Portable Document Format as a trusted means to exchange, preserve and protect healthcare information digitally. The accompanying “Implementation Guide for the Portable Document Format Healthcare” (AIIM BP02-2008, [19]) helps to facilitate the implementation of technical items mentioned in the Best Practices Guide. A recurring point in this document is that the creator of a PDF document should ensure that the consumer uses reading software that is able to reproduce the used PDF features. Even though PDF is actually an ISO standard, many readers are not able to handle the more advanced PDF features like scripting or embedded 3D models.

A general major issue regarding the exchange of medical data is privacy and security. This issue is discussed e.g. in [20], [21] and [22] and solutions are covered by the PDF/H standard. The key approach to overcome respective problems is document encryption. PDF provides the possibility to encrypt its contents using Advanced Encryption Standard (AES) or the RC4 stream cipher and to sign documents digitally [16]. A properly encrypted document cannot be manipulated, making it impossible e.g. for attackers to extract data or to insert malicious elements.

Although there are many use cases for embedding 3D models or other multimedia contents into medical PDF documents [12], this new technology seems not to have found broad acceptance and usage in clinical routine so far. While the use of PDF for the exchange of discharge letters, consultations and excerpts of Electronic Health Records (EHRs) containing plain text and (two dimensional) images is common clinical practice, it is not for multimedia contents and 3D models. Thereby, as shown above, PDF is particularly suitable for presenting and exchanging three-dimensional visualizations, as obtained for example in computer assisted surgical planning.

### Benefits of CASP for Liver Interventions and 3D Visualization

Digital high-resolution imaging (Computed Tomography – CT and Magnetic Resonance Imaging – MRI) enabled the development of CASP systems for patient-specific treatment solutions. Especially in (but not limited to) surgery of the liver, preoperative planning plays an important role in the assessment of resectability and the choice of operative strategy, since the precise understanding of the individual anatomy of a patient is crucial [1], [23], [24]. Virtual resection planning is nowadays an important tool to plan and assess patient-individually optimized surgical strategies [25].

**Figure 2:**
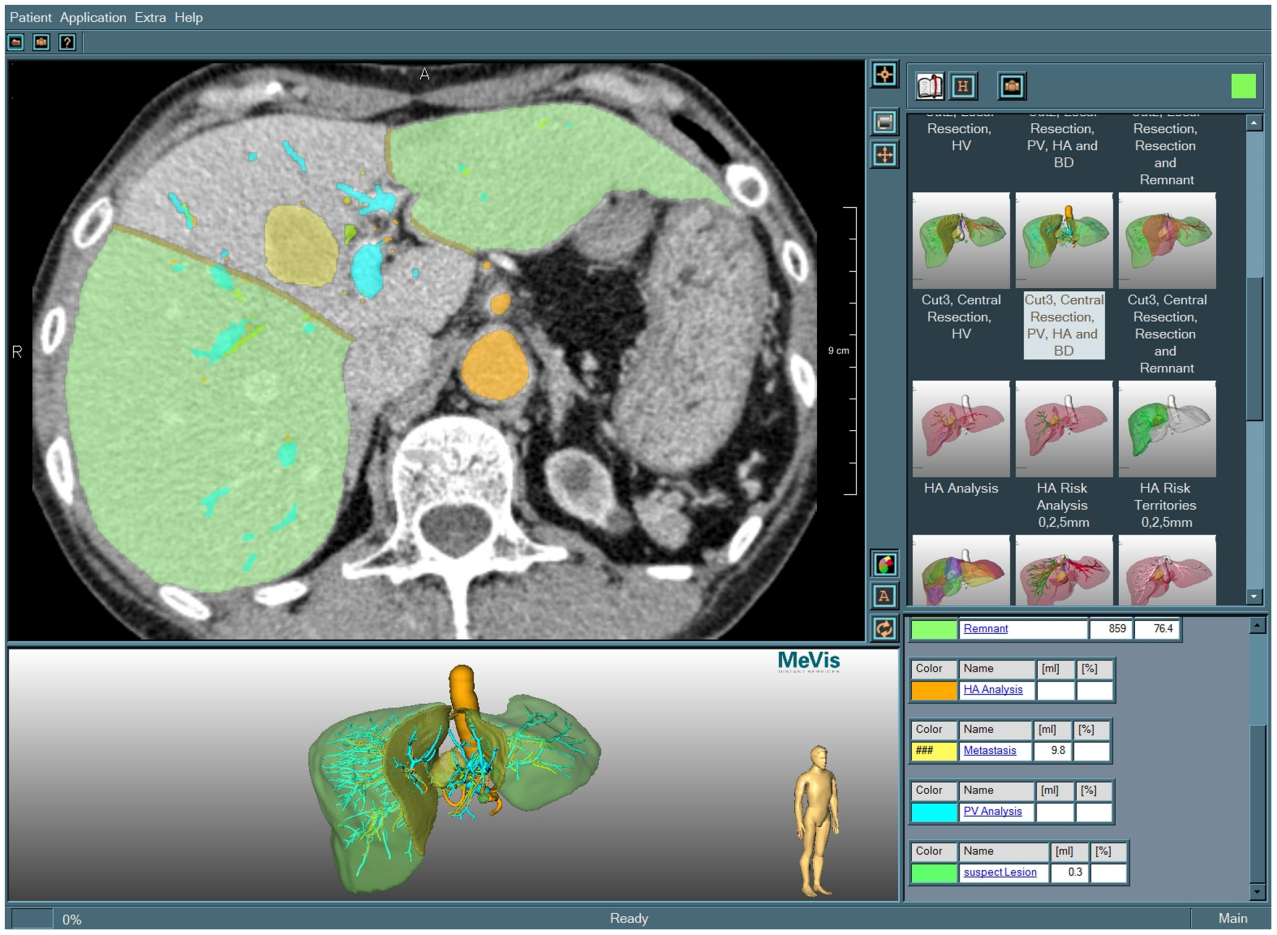
Screenshot of the LiverViewer. The user interface is divided into four parts: CT slices with color overlays (top left), interactive surface shaded objects (bottom left), resection proposal selection (top right) and analysis data (bottom right).

One of the benefits is the emerging 3D visualization (Fig. 1, Fig. 2) that helps to improve the understanding of a patient's individual configuration, thus increasing the subjective confidence for the surgeon and improving the overall results of surgery [26], [27]. A proper visualization of the planning data helps to identify critical structures, and supports the preparation and control of the intraoperative situation [24], [25], [28] – especially since the act of surgery imposes a large cognitive load, limiting the cognitive resources that surgeons can devote to the visualization during the intervention [4]. Another important advantage of 3D visualization is its potential to simplify the discussion of the treatment and surgical procedure between clinicians [3], [24].

### A Telehealth Service for Liver CASP

Liver CASP is a challenging task. It combines a number of image processing steps and requires an appropriate visualization for interactive exploration of results [1], [23].

Using the “LiverAnalyzer”, a software tool developed by the research institute MeVis (nowadays Fraunhofer MEVIS) and approved by the Food and Drug Administration (FDA) for use as a medical device, all the necessary analysis, planning and visualization steps can be performed; the details are described e.g. in [1], [23], [29].

While the LiverAnalyzer enables sophisticated liver surgery planning, it requires trained personnel to operate, which may lead to considerably high costs for in-house application. Therefore, the telehealth service provider MeVis Distant Services AG (MDS) was established, offering liver CASP on a commercial base [30]. Operating on CT and/or MRI image data of an individual patient, MDS creates tailored resection proposals and quantitative analysis results, as well as detailed 3D visualizations of the respective liver anatomy. Since 2004, MDS and the research institute MeVis have processed more than 6000 cases, including cases for scientific studies (e.g. [31] and [32]).

### Motivation

The results of a CASP remotely performed by MDS are primarily produced in the form of proprietary data that can be processed in full extent only by the LiverAnalyzer itself or the “LiverViewer”, a dedicated tool for exploring the respective analysis and planning results that is available for free to all MDS clients [2]. It allows for the visualization in two and three dimensions using overlays onto the original 2D CT or MRI data and surface shaded 3D objects (Fig. 2).

By using the LiverViewer, the attending physician is able to interactively explore and approve all results calculated by the software and to assess the data, e.g. by adjusting the view orientation or by selecting objects of interest.

**Figure 3:**
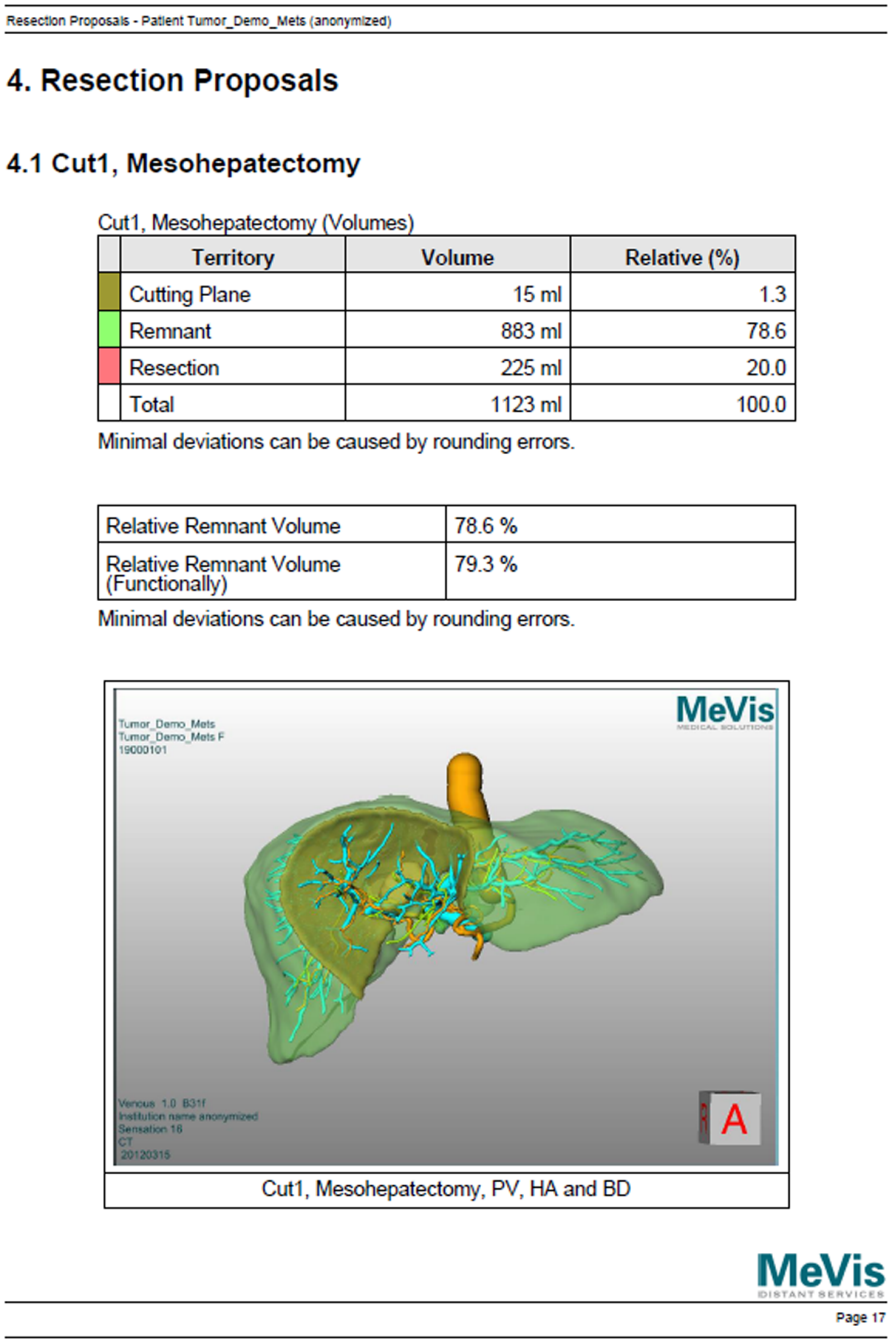
Example of one of the multiple pages from the auxiliary 2D PDF. This page contains tables with volume information (top) and a static screenshot of a resection proposal (bottom).

However, the use of the LiverViewer still requires a considerable amount of training, and exploring all the results in detail is time-consuming. The software needs to be installed on appropriate hardware which might not be available everywhere in a hospital and the volume of the input data may consume up to several hundred megabytes, making it hard to share it. On the other hand, it is not always necessary to unleash the full power of all possible features. Therefore, a reduced but more convenient set of information is provided along with the original planning data: a multi-page PDF file with basic information in written text, tables, screenshots and embedded movies objects (the so-called “2D PDF”, Fig. 3). Tables contain statistical information like territory volumes or tumor burden. Screenshots are single, 2.5D views onto the anatomy or resection proposals. Movies are used to induce a spatial impression by a predefined rotation of the 2.5D view around two axes.

This combination of proprietary LiverViewer data and conventional 2D PDF file is delivered with every planning result – but in purely practical terms it has some shortcomings, though. While the PDF is superior to the LiverViewer as regards the necessity to install dedicated software and the amount of data, it lacks the possibility to fully explore the results in depth. Besides that, the embedded videos, which serve as a workaround for a real interactive 3D exploration, are limited to a fixed set of camera rotations, require a codec and possibly permission to work on the consumer's computer and inflate the PDF file considerably. Furthermore, the embedded screenshots are created by the planning personnel and therefore possibly do not always show what the consumer is interested in. Another disadvantage of the PDF file is that it can consist of dozens of pages, depending on the complexity of the case.

Our goal was to combine the advantages of the interactive exploration as provided by the LiverViewer with the easy exchangeability as provided by the PDF, and to provide the information in a way as user-friendly as possible. A quick, efficient, needs-based and easy-to-use access to the planning data without the requirement to install dedicated software or software extensions or to browse through large documents was another main focus.

## Methods

### Ethics

Although the reports described in the article contain medical data, no medical research was performed at all. All medical data published with this article consists of previously existing, anonymous demonstration data that was freely available beforehand. Therefore, and due to our method of conducting the user survey (see below), according to the Ethics Committee of the Friedrich-Alexander University Erlangen-Nuremberg, an ethical board approval or the inclusion of a data protection instance was not required.

### General Considerations

Our solution for exchanging the 3D CASP data had to provide the following main features: (A) an interactive visualization of 3D data in real-time and (B) a high level of privacy and security. In practical terms, independence from proprietary/dedicated software and a small to moderate file size were desirable.

With special respect to (B), the Portable Document Format with embedded 3D models turned out to be the best medium after considering alternative solutions (Adobe Shockwave & Flash, Apple Quicktime VR, Virtual Reality Modeling Language 97, Table 1). It was decided to use this format, since it was the only one that was available for free and for all major operating systems (Windows, MacOS, Linux), that could be encrypted and that provided the necessary interaction capabilities. The latter was (besides encryption) a requirement of highest importance since interaction is the key to yield the full potential of 3D data [4].

**Table 1:** Overview of considered technologies.

Requirement	Shockwave	Flash	Quicktime VR	VRML ‘97	3D PDF
Open standard	no	no	no	yes	yes
**Encryption**	**no**	**no**	**no**	**no**	**yes**
3D surface mesh models	yes	(yes)^1^	no	yes	yes
Movies/2D images	yes	yes	yes	no	yes
Standalone player without license cost	no	no	yes	no	yes
Player available for Windows	yes	yes	yes	yes	yes
Player available for MacOS	yes	yes	yes	yes	yes
Player available for Linux	no	yes	(yes)^1^	yes	yes
**Player with built-in interaction**	**no**	**(yes)** 2	**(yes)** 2	**(yes)** 2	**yes**

(yes) in brackets indicates some minor limitations. Important features are emphasized in bold font.

1 needs plug-in or additional efforts |

2 limited functionality.

In order to avoid any human-induced errors during the 3D PDF creation, a fully automatic process was strived for, and with respect to the 510k clearance of the LiverAnalyzer, no modification of that existing planning software was allowed. Therefore, two software modules were created: a mesh export module for generation of the visualization models and a report generator for assembling of the final PDF.

The latter fully automatically collects all data, creates the necessary scripting code and compiles the final PDF document within a few seconds. Since this tool is tailored to our specific use case, a more detailed description is not within the scope of this article.

### Creation of the Surface Models

For the visualization of the (intra-)hepatic structures, a volume rendering solution as described in [15] or [33] would have been a possible solution, but for therapy planning, model-based, illustrative techniques are a better choice. Not only do they enhance the shape perception (which plays an important role in the distinction of objects [4]), but also do they allow for encoding additional information (like segment assignment or safety margin classification) on the structures' surfaces [34].

The LiverAnalyzer is based on MeVisLab (http://www.mevislab.de/), a framework for medical image processing [35]. Due to the modular design of MeVisLab, it was possible to attach the mesh export module to the LiverAnalyzer without interfering with the existing implementation. The mesh export module takes intermediate data (e.g. segmentation masks or vessel trees) that fall of during the planning process and uses them to create surface meshes of all relevant structures (the liver, tumors, vessels, perfusion territories and so on) in the Universal 3D (U3D) format. U3D is a standardized binary file format [36] and one of the 3D object file formats that can be embedded into PDF files. It contains all necessary information to completely describe a 3D scene graph. The U3D format and its generation are described in detail in [22].

### Creation of the User Interface

**Figure 4:**
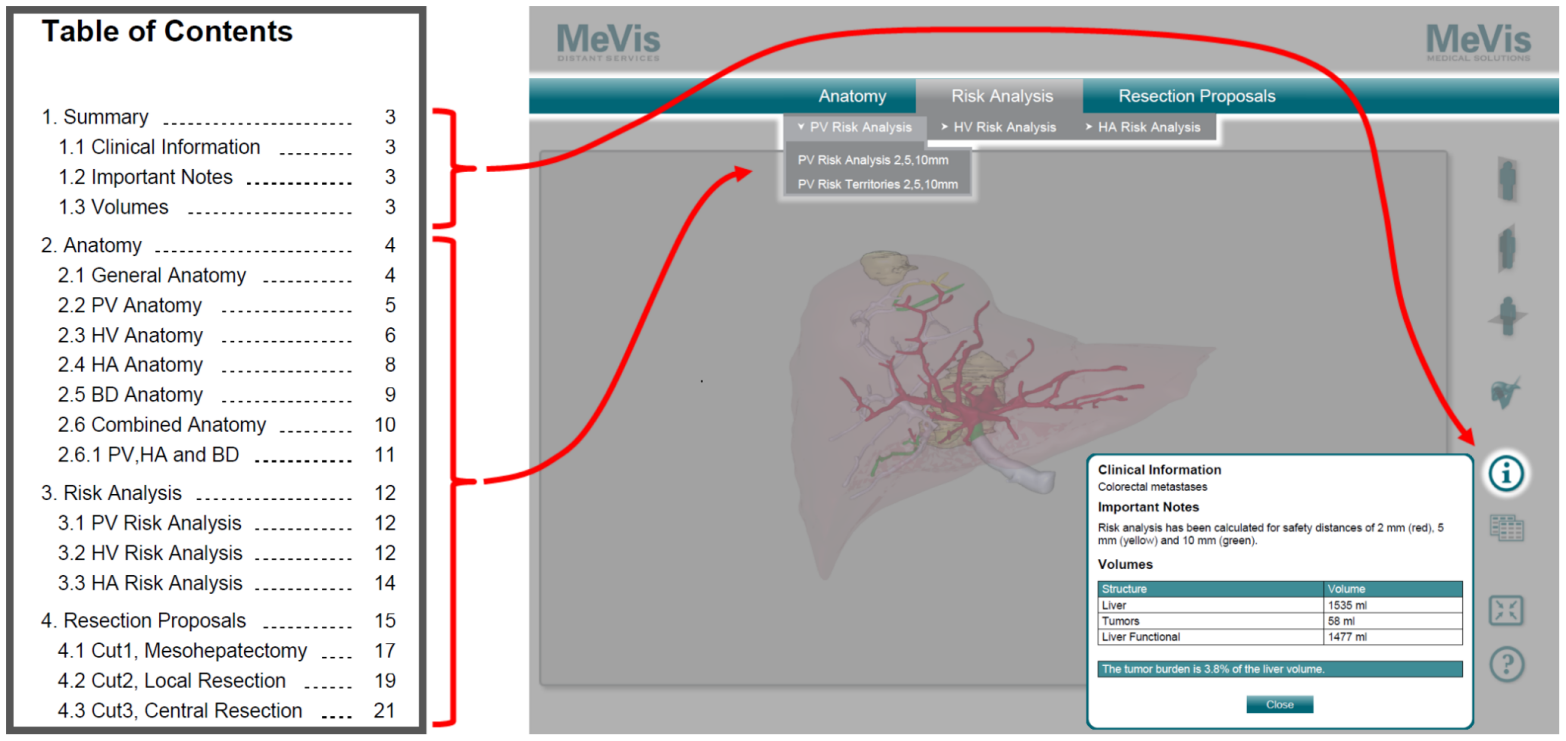
Conversion of the multi-page chapter structure of the 2D PDF. The chapter structure of the conventional 2D PDF (left) was converted into a single-page menu structure in the 3D PDF (right). Contents of the summary chapter can be accessed by the “i”-button, which opens an overlay window. Contents of the remaining chapters can be accessed by respective drop-down menu items.

In a typical CASP case, several resection proposals are planned so that the surgeon can compare the different approaches [1], [23]. Switching between the different proposals and views should be as easy as possible and the navigational steps needed to get certain information should be reduced to a minimum [4]. Therefore, the videos and screenshots in the precursor 2D PDF were not simply replaced by 3D models. Instead, a one-page, application-like design was chosen: the goal was to make all information and all anatomic models available on a single page and accessible by means of a selection menu instead of multiple pages with partial information on each. Nevertheless, especially regular 2D PDF users should quickly become familiar with the new layout. For this reason, the settled chapter structure of the 2D PDF was transformed into a drop-down menu structure for the 3D PDF (Fig. 4). General case information was made available through an overlay window opened by a dedicated “information” button to make it accessible regardless of the menu selection. Other overlay windows were chosen to display supporting information (like color legends, segment volumes or other statistical data) belonging to the recently selected and visible set (“collection”) of objects in the 3D scene window.

To prevent the consumer of the report document from getting lost in the depth of a complex 3D scene, three navigation buttons for default coronal, sagittal and axial views were integrated. Furthermore, two comfort functions were added: a button to switch between windowed and full screen view mode and a built-in help feature.

Bringing this user interface to life required the usage of scripting. PDF fully supports JavaScript (version 1.6) as cross-platform scripting language and means for interacting with GUI controls and multimedia content [37]. Since every case report is unique (depending on the patient's anatomy and the client's request), the necessary scripts as well as the GUI elements for the menu structure need to be generated on-the-fly while the report is compiled by the report generator.

### Rendering Issues

Internal reviews of early report prototypes unveiled three issues as regards the visibility of objects due to weaknesses of the 3D scene renderer of Adobe Reader: (A) multiply stacked transparent objects could lead to renderings in which it could be hard for a beholder to distinguish them, (B) in seldom cases small tumors could happen to be obscured by overlying (though transparent) objects in adverse viewing angles and (C) tumors that were segmented from a single slice of the original CT dataset were not displayed, although the model data was exported to U3D.

**Figure 5:**
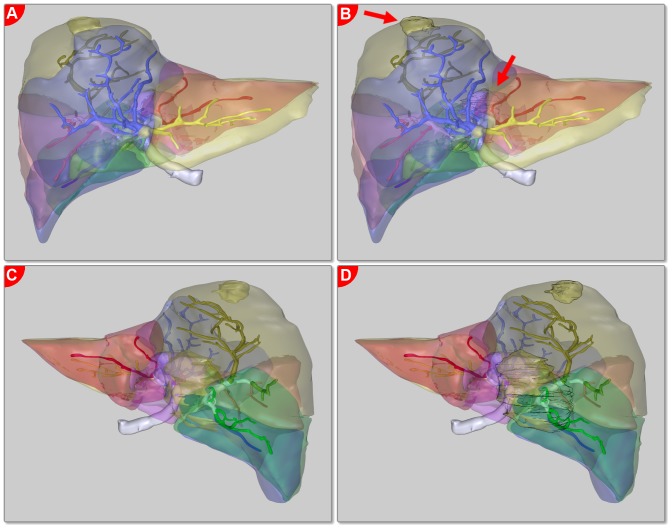
Emphasis of tumors by accentuating their contours. Four projections of the same scene with tumors not emphasized (left, (A) and (C)) and emphasized (right, (B) and (D)) in two different projections (anterior-posterior, top, (A) and (B) and posterior-anterior, bottom, (C) and (D)). Emphasized tumors can be perceived better, even if they are lying behind vessels and territory borders (arrow marks).

Therefore, the initial report concept was revised to overcome these issues. A button was added to the report GUI that allows for switching off and on certain transparent objects that are not absolutely necessary (e. g. the liver surface) to mitigate (A). Issue (B) was solved by application of a different rendering mode for tumors and other risk structures: instead of only rendering them like the other objects in “solid” rendering mode (a PDF term), the contour was enhanced (“solid outline” rendering mode), giving them a silhouetted, cel shaded look and emphasizing them compared to the other objects. This improved the overall visibility of small risk structures [4] and as a positive side effect, their occlusion by overlying objects was eliminated (Fig. 5).

Issue (C) required a workaround which engages in the planning phase. Since the rendering engine of Adobe Reader is provided by Adobe, it is not possible to modify it. Changing the model data or the rendering options would also not have solved the problem. Instead, in case of one-slice tumors, the planning is performed on subsampled data so that the tumor appears on more than one slice.

The modified report version with fixed issues (A) and (B) was then evaluated and rolled out to the clients.

### Evaluation

Although the software modules for generation of the U3D model data and the report itself did not interfere with the previous implementation of the LiverAnalyzer and thus did not jeopardize the 510k clearance, a full formal software test cycle needed for an FDA approval was carried out and documented. No software errors were found.

The evaluation of the 3D PDF report produced by our software was carried out on two levels: accuracy of the content and user experience.

#### Accuracy Evaluation

For the first task, the 3D object collections of the 3D PDF as rendered by the Adobe Reader were compared with the original 3D visualization of the planning results in the LiverAnalyzer for 16 cases (8 living donor liver transplantation (LDLT) planning, 7 tumor resection planning, 1 tumor burden assessment visualization without planning) by two experts, that both had experience with several hundred liver CASP cases.

The LiverAnalyzer visualization was chosen as “gold standard” since it was the basis for the 510k clearance of the planning software and because it contains exactly the same rendering engine that was used for the LiverViewer provided to the clients. Except the missing one-slice tumors, both experts found no differences as regards the configuration and spatial distribution of vessels, territories, tumors or any other planning related objects (like cutting planes or graft volumes). Since no differences were found at all, no inter-rater agreement measure was calculated.

#### User Experience Evaluation

After more than 1000 planning cases that have been reported in clinical routine using the 3D PDF, an online user survey was carried out to evaluate the user experience as regards the acceptance, perspicuity, efficiency and ease-to-use. We did not start the evaluation immediately after the first 3D reports had been rolled out. Instead, we waited until the report was established in clinical routine so that the users could gather enough experience to profoundly assess this new technology.

Only users (i.e. medical staff, no patients) who had a known personal e-mail address and who had received at least one 3D PDF report between January 2012 and September 2013 were invited to participate voluntarily over the course of October 2013. Although a larger user group could have been invited, thus possibly resulting in a larger sample size, we considered it dubious to use nonpersonal, institutional e-mail addresses or to simply invite all theoretically possible users, regardless of whether they had actually ever got a 3D PDF report.

The invitation e-mail comprised the purpose of the survey, the length and time of the survey and the information that all answers were recorded and processed completely anonymously. The starting page of the online survey (see S1 File) again informed the participants about the purpose, the estimated effort and the anonymous participation. Since the interviewees were only invited and since no incentives were offered, participation itself was considered as consent. The survey was hosted by Qualtrics (http://qualtrics.com/).

The authors were at no time in direct contact with the respondents or with any of their personal data (including the e-mail addresses). The survey was conducted completely anonymously from the perspective of the authors. They only got the results provided by Qualtrics. On the other hand, MDS has not received any data that would allow a link between the results and the mail addresses.

The survey was designed in a way that the respondents could cancel their participation at any time and that any question could be skipped. Partial responses were not considered for analysis.

The questionnaire (see S1 File) was constructed under guidance of an experienced psychologist and comprised 5 demographic questions and 9 questions regarding the functionality and the usage of the 3D PDF and its precursor report formats (2D PDF and LiverViewer). One of the key questions was the question about the frequency of use of the different report formats. Depending on the answer to this question, the respondents were directed to either a “3D PDF user” path or a “3D PDF non-user” path. Participants were classified as “non-users” if they selected “Never” or “I do not know this” when asked how often they use the 3D PDF.

The questionnaire was completed by a shortened version of the User Experience Questionnaire (UEQ, http://www.ueq-online.org/) published by Laugwitz et. al. [38]. Only the scales “attractiveness”, “perspicuity”, “efficiency”, and “novelty” (18 items in total) of the UEQ were queried; the scales “dependability” and “stimulation” (8 items in total) were excluded. The arrangement of the included items was left unchanged. We used the UEQ because it is short and because the scales show high internal consistency, resulting in high values for Cronbach's alpha [38].

The questionnaire was initially designed in German language and translated to English with assistance of a native English speaker. The items of the UEQ were already available in German and English from [39] and [40].

The statistical analysis was performed using IBM SPSS Statistics 20. If responses from free response options could doubtlessly be mapped to a predefined option, then the answers were reassigned by us to the predefined option (e.g. free answer: “transplant surgeon” → predefined option: “surgeon”).

The UEQ was evaluated using the template provided by its authors [39], [40]. Details of this procedure are described in [41].

Detailed results of the evaluation are provided in the next chapter.

## Results

### The 3D PDF Report

Our solution is an interactive 3D PDF report of liver surgery planning results that acts more like an application (“PDF App”) than like a typical PDF document. Two fully functional example reports with demo data for oncology [42] and living donor liver transplantation [43] are available for download.

**Figure 6:**
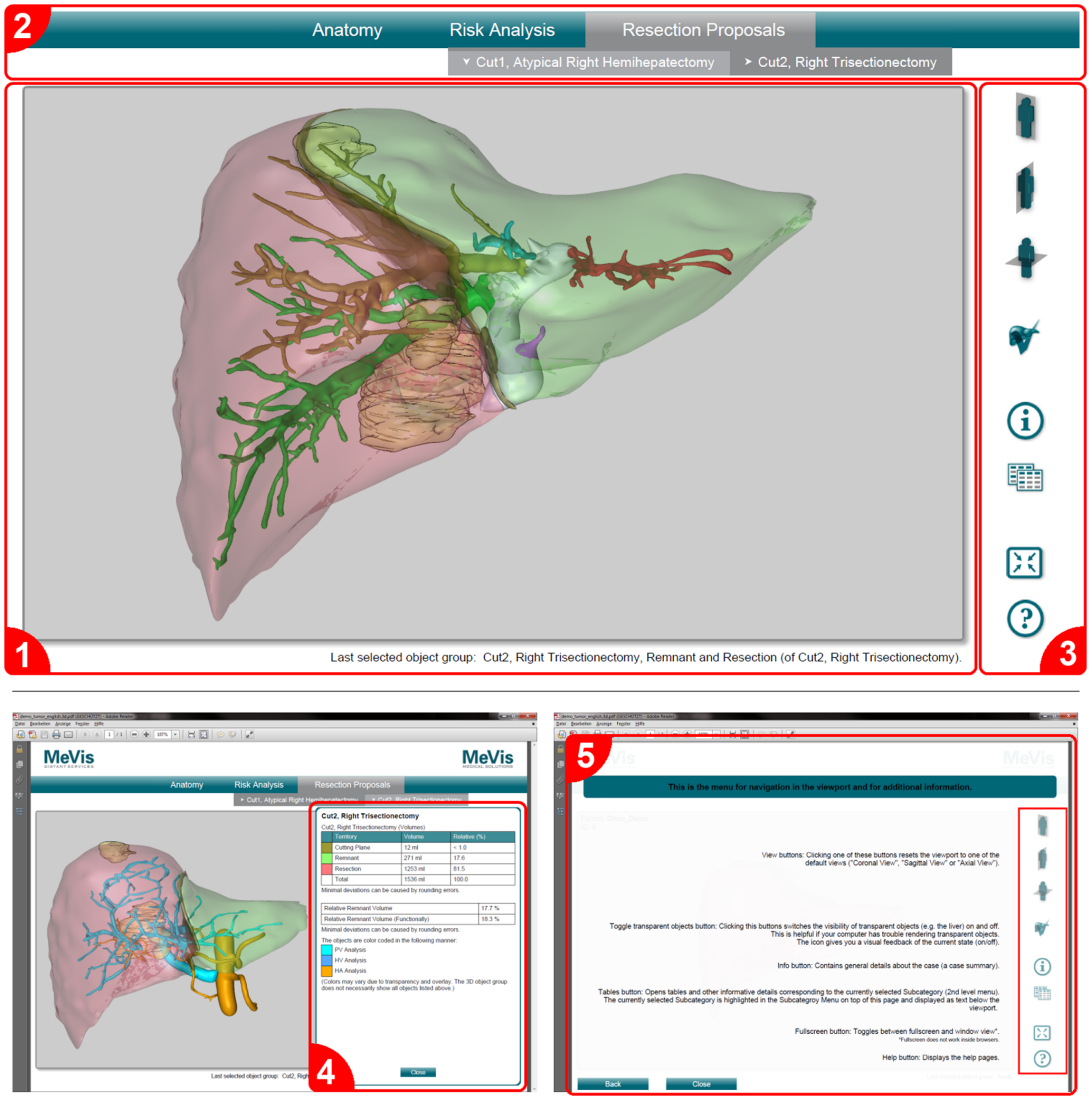
User interface of the 3D PDF report. (1) 3D viewport with scene information at the bottom, (2) drop-down menu for selection of 3D objects, (3) navigation menu, (4) overlay window for resection-specific information and (5) built-in help.

All results are accessible from one single and clearly arranged page without the need of scrolling. The user interface (Fig. 6) mainly consists of three parts: a 3D viewport that displays the interactive 3D scene, a drop-down menu that allows for a convenient selection of the different object collections and a navigation menu for accessing default views, statistical data, annotations and comfort tools. An additional minor part of the UI displays information about which object collection is currently displayed in the 3D scene.

To properly display this report, Adobe Reader 9.1 (or a later version) is required. Any fully compatible PDF viewing software would work as well, but currently there is no alternative available that completely supports the PDF standard in full extent (especially regarding embedded 3D models and scripting).

Using the Adobe Reader, the objects in the 3D scene can be explored by rotating, panning and zooming. In case that a user gets lost inside the depth of a scene, three pre-defined views that show the full scene from standard medical directions are available through quick access buttons. The object collections can be selected from a drop-down menu as known from many software applications. General information about the case or the currently selected object collection, annotations made by the planning personnel and statistical data can be retrieved from pop-up overlay windows (Fig. 6).

To ensure the integrity of the embedded health care data as demanded by [44], the PDF document is encrypted with a long, pseudo-randomly generated unique password. The applied PDF security properties only allow to print the document and to use the interactive form fields, but not to modify anything of the content. As a positive side effect, the latter also prevents possible attackers from inserting malicious code into the embedded interactive elements.

The 3D PDF report complies with the PDF/H standard [17]. The users have been informed that the Adobe Reader 9.1 or later has to be used to ensure that all features can be accessed properly.

Finally, the report has a built-in help function that explains the basic functionality and is accessible at any time (Fig. 6). Table 2 comprises a summary of all features of the 3D PDF compared to the 2D PDF and the LiverViewer.

**Table 2:** Feature overview of the report data formats.

Feature	LiverViewer	2D PDF	3D PDF
Interactive exploration of anatomy and planning results	yes	no	yes
Printing of user-selected views	yes	no	yes
Tables and informational text	yes	yes	yes
Annotations and comments from by the planning staff	no	yes	yes
All informations on a single page/screen	yes	no	yes
All informations in a single file	no	yes	yes
Independence from dedicated software (except Adobe Reader)	no	yes	yes
Independence from video codecs	yes	no*	yes
Use without training	no	yes	yes
Simple exchange with others	no	yes	yes
Availability for all platforms (Windows, MacOS, Linux)	no	yes	yes
Protection from manipulation	no	no	yes
Display of original radiological data	yes	yes*	no
Rendering engine under control	yes	no	no
Overall data file size	large (>100 MB)	small (∼1.5 MB) to moderate*	moderate (∼10 MB)

*  =  with embedded videos.

### Results of the User Survey

#### Participants

A total of 138 users from all over the world was invited to the survey; 30 users (21.7%) from 14 countries in Europe, Asia, Africa and North America participated.

Only one respondent who has described his or her position as a “research scientist” answered that he or she does not use the 3D PDF (nor the 2D PDF) at all, because he or she does not know these formats (instead, the LiverViewer is used). Therefore, this response was not considered further. 29 respondents (96.7%) use the 3D PDF at least rarely; 3 of them were excluded from the analysis since they did not fill in the questionnaire completely. This results in 26 users, which were included in the evaluation.

The age distribution of these 26 users was as follows: 26.9% were 30–40 years old, 53.8% were 40–50 years old and 19.2% were more than 50 years old. 84.6% of the respondents said that they were surgeons, 15.4% radiologists.

A large group (76.9%) of the respondents uses the MDS service for oncological cases with an average of 15 cases per year. LDLT cases are handled by 57.7% of respondents with an average of 18 cases per year. For 19.2%, follow-up evaluations are a reason to consult MDS (average 34 cases per year). Additionally, 11.5% of the users have specified other service requests with an average of 28 cases per year. The respondents work primarily (76.9%) in medium-sized hospitals with 50-200 liver surgery cases per year.

#### Frequency of Use

The frequency of use of the different report formats was assessed with the question “How often do you use each report format?”. For each category the users could choose between six possible answers: “Always”, Often”, “Sometimes”, “Rarely”, “Never” or “I do not know this”.

**Figure 7:**
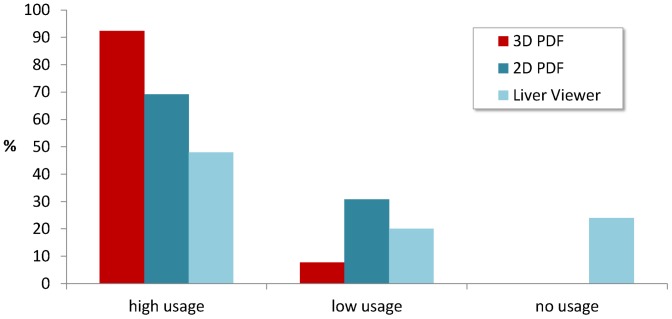
Usage rates of the three report formats 3D PDF, 2D PDF and LiverViewer. Almost all respondents (92.3%) use the 3D PDF often or always (“high usage”). The remaining respondents use it rarely or sometimes (“low usage”).

Nearly two-thirds (65.4%) use the 3D PDF always, and almost every respondent (92.3%) uses it often or always. For further evaluation, both pairs of options “always”/“often” and “sometimes”/“rarely” were added together, resulting in users with high and low usage rates. As depicted in Fig. 7, the 3D PDF is most frequently used, followed by the 2D PDF. While 2D PDF and 3D PDF are both known by all users, the LiverViewer is not known by 7.7% of the respondents. 52.2% of the remainders are high-frequent users of the LiverViewer and 21.7% are low-frequent users while 26.1% never use the LiverViewer.

Initially, we were also interested, if low-frequent users of the 3D PDF do use the LiverViewer more frequently instead. Since 92.3% are high-frequent users of the 3D PDF, this question became obsolete.

#### Kind and Setting of Usage

Almost all respondents (92.3%) use the 3D PDF for their decision about the surgical strategy. At least half of the users apply the 3D PDF for deciding whether surgery is performed (53.8%), for personal or mental preparation for surgery (53.8%), support during surgery, for discussions with colleagues (65.4%), for education or rather training (69.2%), or for case presentations for instance at conferences (53.8%). Only 30.8% of the respondents use the 3D PDF for patient information and 26.9% use the 3D PDF for discussions in the tumor board. One respondent uses the 3D PDF for navigation.

The possibility to exchange the 3D PDF with others is used by 76.9% (70.0% by e-mail and 35.0% by a hardware storage medium). 88.5% display the 3D PDF in the operating room using the following media: paper printouts with pre-defined views (11.5%), paper printouts with customized views (15.4%), PC monitors (42.3%), wall-mounted large screens (15.4%), notebooks (34.6%), and tablet PCs (11.5%).

#### User Assessment of the 3D PDF Features and Usability

The participants were asked to rate the statement “The following functions and characteristics of the 3D PDF are useful.” for several features offered by the 3D PDF. Each statement could be rated within an ordinal scale between 4 and 1 (4: “Agree”, 3: ”Somewhat agree”, 2: “Somewhat disagree”, 1: “Disagree”). For each statement, the mean value and the standard deviation were calculated (Table 3). All functions of the 3D PDF were rated to be useful (all mean values >3.0).

**Table 3:** User assessment of the usefulness of the characteristics and features of the 3D PDF.

Features	N	Mean	Std. Dev.	Agree	Somewhat agree	Somewhat disagree	Disagree
				(4)	(3)	(2)	(1)
*Interactive Zooming and viewing of the 3D model from all directions*	26	3.7	.5	76.9%	19.2%	3.8%	0%
*Interactive Selection of different views and resection proposals*	26	3.6	.6	61.5%	34.6%	3.8%	0%
*Interactive show/hide of the liver parenchyma*	26	3.4	.8	57.7%	30.8%	7.7%	3.8%
*Tables and informational text for case summary*	26	3.5	.6	53.8%	38.5%	7.7%	0%
*Tables and informational text for the currently selected 3D view*	25	3.5	.7	56.0%	36.0%	8.0%	0%
*Summary of all information in one page (without scrolling)*	25	3.4	.7	52.0%	40.0%	8.0%	0%
*Grouping of all information in one file*	24	3.5	.7	66.7%	20.8%	12.5%	0%
*Independence of pre-installed dedicated software (except Adobe Reader)*	23	3.6	.6	60.9%	34.8%	4.3%	0%

Most of the respondents agreed or somewhat agreed that the 3D PDF is easy to use while opening or viewing (92.3%) and while exchanging with others (88.5%).

Additional features that have already been identified by us as subjects for future extension were rated worthwhile by the respondents as well: 92.3% agreed or somewhat agreed that the overlay of planning results with original radiological data would be a useful extension. All participants (100%) agreed or somewhat agreed, that it would be useful to integrate a self-planning feature to create individual resection plannings.

#### User Experience Investigated by the UEQ

The results of the scales attractiveness, perspicuity, efficiency and novelty from the UEQ can be found in Table 4. The users rate the 3D PDF positive at all scales (note that mean values >+.8 denote a good rating and mean values >+1.5 denote a very good rating [39], [40]). The best score was reached for the attractiveness scale (M = 1.872). Similarly positive results were found for the scales perspicuity (M = 1.673) and efficiency (M = 1.644). The relatively lowest, but still very high score was reached in the novelty scale (M = 1.375).

**Table 4:** Results of the UEQ.

Scale	Mean	Std. Dev.	Confidence	Confidence Interval			Cronbach's α
**Attractiveness**	1.872	.928	.357	1.515	–	2.228	.94
**Perspicuity**	1.673	1.029	.396	1.278	–	2.069	.84
**Efficiency**	1.644	.917	.352	1.292	–	1.997	.72
**Novelty**	1.375	1.087	.418	.957	–	1.793	.74

Confidence intervals were estimated with a p-value of p = .05 per scale.

The internal consistency (Cronbach's α) in our survey was good for the scales perspicuity (α = .84), efficiency (α = .72) and novelty (α = .74) and very good for the scale attractiveness (α = .94).

## Discussion

### Comparison of the 3D PDF with the Previous Solution

Our goal was to combine the advantages of interactive exploration with the platform-independent display capabilities and simple exchange of the data. This goal was fully reached; the new 3D PDF offers many advantages over the pre-existing solution (Table 2). More than 75% of the users take advantage of the possibility to exchange the 3D PDF with others.

The only feature that is provided by the precursor report formats but not the 3D PDF is the possibility to display the original radiological data that was used as the base for planning (Fig. 2, top left). The user survey shows that this would be a useful feature, and technically, it would have been feasible to integrate this feature into the 3D PDF as well. On the other side, this would require to integrate the complete radiological dataset into the PDF, which would result in a vast inflation of the file size. Since one of the main goals for the 3D PDF was to keep it easy to exchange, the integration of original data was intentionally discarded.

### Suitability of 3D PDF for Clinical Routine

Using Adobe Reader, almost any modern off-the-shelf computer is capable of visualizing even complex results of CASP without the need to install additional software (or extensions to existing software) and without any license cost.

**Figure 8:**
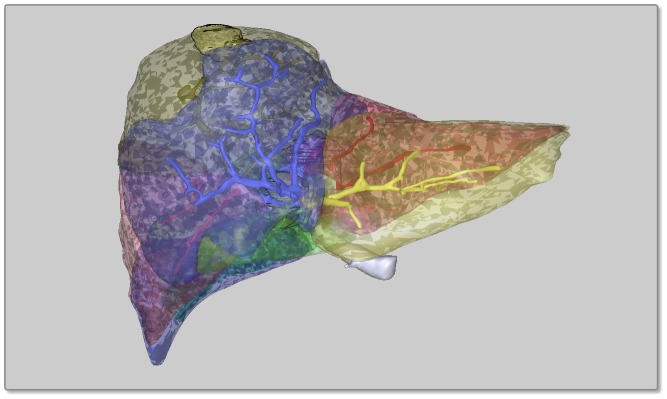
Rendering artifacts. These tessellation artifacts have been observed on low-end graphic boards in MacOS hardware.

However, there are two major drawbacks that need to be mentioned. First, a rendering flaw on low-end graphic boards in MacOS hardware has been observed (Fig. 8). Adobe Reader for MacOS seems to have trouble with multiple transparent surfaces that need to be rendered on top of each other. This is possibly due to a “speed-over-quality” strategy of the software-based renderer that pitches in if no hardware rendering solution is available since rendering of transparent objects is a challenging task even for modern software [45]. The same effect may happen on other platforms but has not been reported yet. Users experiencing this tessellation effect need to move to other hardware or (in our use case) to fall back to the 2D PDF or LiverViewer.

Since our solution builds on the standard version, the rendering engine used to display the surface models in Adobe Reader cannot be influenced. On the other hand, Adobe Reader is currently the only suitable tool for displaying 3D PDF. As a consequence, during the design of a medical 3D PDF report, care must be taken to identify all possible rendering artifacts and issues that may deteriorate the information that shall be displayed.

Additionally, designers of a 3D PDF are limited to the possibilities the PDF specification provides. For example, the effect of using contour enhanced rendering to mitigate the sub-optimal visibility of stacked transparent objects as described earlier (Fig. 5) could possibly be strengthened by using suggestive contours [46] instead. Unfortunately, the current PDF specification did not envisage this option and therefore the designers must make the best out of the realities.

The second issue is the lack of suitable PDF reading software for tablet computers. While the Adobe Readers for Windows, MacOS and Linux fully support the PDF standard, the current versions for iOS and Android do not. Hence our 3D PDF reports can be displayed as expected on x86/64 based tablet computers with Windows operating system, but not on the popular Apple iPad or on devices that use ARM processors. All PDF readers that are available for the latter two platforms are not capable of displaying embedded 3D models. The result of the user survey shows that there is a considerable demand for tablet viewing software.

An additional minor issue is security considerations, but this is not a specific problem related to embedded 3D models and therefore a detailed discussion is out of scope of this article. The most important aspect is that our solution complies with the PDF/H standard and therefore does not introduce a new risk. Compared to embedded videos, it even reduces the risks since no external resources (like video codecs) are required. Furthermore, most concerns in this regard can easily be solved if the respective 3D PDF documents are encrypted as described above since rigorous encryption makes it impossible for an attacker to insert malicious elements or JavaScript code into the document.

However, even if a 3D PDF report with embedded JavaScript is not an actual threat, it might be considered to be one. Therefore, IT environments in a medical context may have very restrictive policies as regards displaying these types of documents and respective functions (especially the execution of JavaScript) might even be deactivated. Version X (10) of Adobe Reader introduced the “Protected Mode” (also called “Sandboxing”, [47]), which lets the Software run in an isolated environment and which prevents malicious activities from attacking the system it is running on. Therefore, while using version 9.1 or later is required to display a 3D PDF with embedded JavaScript code, Version X or later is recommended in a safety-critical environment since it allows for using interactive elements without violating security requirements.

### User Reception

The initial acceptance of the 3D PDF report was very good. A few users did not immediately recognize that the new format allowed for direct interaction with the objects in the viewport or were unassertive since they did not expect a PDF file to be dynamic. But after the first uncertainty, they quickly became familiar with all features thanks to the well-known GUI principle of drop-down menus.

Our user survey revealed that the 3D PDF is used by almost every participant (surgeons as well as radiologists) for a wide range of applications that go beyond the primary purpose of transmitting the MDS planning results to the customers. Only one respondent does not use the 3D PDF since he or she does not know the 3D PDF. Maybe this is because of his/her role as “research scientist” who absolutely needs the full functionality of the LiverViewer. It remains to consider that other non-users may simply have not responded, but since the invitation letter has only referred the various reporting formats in general, we consider this possibility not to be highly probable.

The users assess the features and characteristics to be useful and they have found new fields of application that were not foreseen by us. The results of the UEQ back this up: the general impression of the 3D PDF is very good and it is easy to understand as well as to get familiar with it. Furthermore, the UEQ results show that our 3D PDF has an organized interface and that it can be used efficiently. Finally, the users assess it to be innovative.

### Benefits for Medical Staff and Further Use Cases

We were able to proof that using PDF with embedded 3D models for reporting and sharing liver CASP results from a telehealth service is feasible and well accepted by the target audience. Since PDF is a widely accepted file format and dedicated to the exchange of electronic documents, our specific solution is not only suitable as a carrier vehicle for transmitting the planning results. The report documents are also used for secondary purposes like tumor board discussions or case presentations at conferences. However, the presented technology is neither limited to the liver nor to CASP of course. Basically every medical discipline and use case that can benefit from 3D visualization and that needs respective information exchange is a candidate for the usage of 3D PDF.

For example, the assessment of facial fractures is often difficult due to the complex anatomy of the jawbones and the large amount of possible fracture patterns [48]. 3D visualization can facilitate evaluation of CT data in patients with facial trauma by displaying the spatial relationship of the different anatomical and pathological structures such as fracture gaps and fracture fragments. In [48], Rodt et. al. have shown that surface rendering has a diagnostic benefit and therefore is superior to volume rendering. 3D PDF provides a perfect means to exchange such renderings using an industry standard file format, e.g. for teleconsultation.

Another example is angiography. Just like in the liver, the complex depth structure of angiography datasets makes spatial cognition one of the most challenging tasks during their exploration [49]. Three-dimensional visualizations of e.g. cerebral vessel structures are helpful for diagnosing diseases and especially the treatment of a cerebrovascular disease requires a good spatial comprehension of the respective vessel configuration [50]. All the technical methods to improve non-interactive visualization as described e.g. in [49] become obsolete if interactive 3D PDF was used instead.

Furthermore, a lot of other use cases are conceivable: fiber tracking in neurology/neurosurgery, bronchial branching patterns or vectorcardiograms, to list just a few.

Besides the pure integration of medical 3D models, we have also shown that it is possible to create PDF documents that look and feel like applications (“PDF Apps”). The resulting enhanced interaction options are enablers for facilitating the handling of even very complex medical informations since they let the user select which information shall be displayed and in which depth. Several authors have shown that interactive 3D visualization improves anatomy learning [51], [52] and since 3D PDF is an advanced development of the technology used by them that provides much more control for the users, it could be a very good tool for training of medical students or future specialists.

### Benefits for Patients

Apart from the improved prognosis of treatment, patients may also benefit from the 3D PDF on a different level. It is an ethical obligation and thus a standard of care, that every invasive intervention requires the patient's informed consent [53]. On the other hand, patient anxiety and dissatisfaction results from uncertainty and lack of information or explanation. This is often due to the doctor's language, which may be hard to understand for the layperson [54]. Hence it can be very difficult for the physician to impart the necessary understanding of a planned procedure, its risks and future consequences without using his domain-specific language [53]. Therefore, understandability, readability and ease of access to materials are prerequisites for a good patient education, as e.g. required by the Joint Commission on Accreditation of Healthcare Organizations (JCAHO) as a condition of accreditation [55]. Several studies have shown that patient education supported by multimedia and 3D visualization leads to better knowledge, satisfaction and reduced anxiety on the patient's side, though not taking more time for the patient-physician interaction [53], [56]. In [55], Fox et. al. introduce a “best practices model” for interactive, multimedia based patient education. 3D PDF is a means to immediately and simply implement five of these eight best practices and in our scenario a way to present even complex liver surgery cases in a way that patients will understand their individual case more easily than without multimedia support.

Our survey results show that only about one third of the respondents use our 3D PDF for patient education. We do not know the reasons, but we see here further potential to use the 3D PDF even more beneficial.

## Conclusion

We presented the first clinical routine application of highly detailed 3D surface mesh models integrated into automatically generated and fully interactive PDF files for the presentation and exchange of computer assisted surgery planning results in liver surgery. The producing software has been validated using a full regulatory protocol.

We demonstrated that the usage of 3D PDF for reporting and sharing CASP results is feasible and well accepted by the target audience. Although the specific software tool developed for the creation of our 3D PDF reports is applicable for our use case only, the general methodology of using interactive PDF with embedded 3D models and scripting as a means for exchanging complex medical information is not: our results open the door for a wider use of this new technology, since the basic idea can and should be applied for many other medical disciplines and use cases.

## Supporting Information

S1 File **3D PDF report examples.** A PDF file containing further screenshots from 3D PDF reports described in this article. This file also contains download links for complete 3D PDF reports.
(PDF)

S2 File **The questionnaire (English version).** PDF version of the questionnaire made of screenshots from the online survey tool.
(PDF)

S3 File **Original raw data of the survey.** XLSX file containing the original raw data of the survey.
(XLSX)
