# Correction: Application and Evaluation of Interactive 3D PDF for Presenting and Sharing Planning Results for Liver Surgery in Clinical Routine

**DOI:** 10.1371/journal.pone.0120158

**Published:** 2015-03-19

**Authors:** The PLOS ONE Staff

The legends for S1 File and S2 File are incorrectly switched. Please view S1 File and S2 File with their correct legends below.

## Supporting Information

S1 File The questionnaire (English version). PDF version of the questionnaire made of screenshots from the online survey tool.
(PDF)

S2 File 3D PDF report examples. A PDF file containing further screenshots from 3D PDF reports described in this article. This file also contains download links for complete 3D PDF reports.
(PDF)
