# Supplementary material for: Application and Evaluation of Interactive 3D PDF for Presenting and Sharing Planning Results for Liver Surgery in Clinical Routine
Source: PLoS One. 2014 Dec 31;9(12):e115697. doi: 10.1371/journal.pone.0115697 (PMC4281211; doi:10.1371/journal.pone.0115697)
Supplement: S1 File — 3D PDF report examples. A PDF file containing further screenshots from 3D PDF reports described in this article. This file also contains download links for complete 3D PDF reports. (PDF) [file pone.0115697.s001.pdf]

Part 1 – for all interviewees.

qualtrics.com

English

Fraunhofer MEVIS | MeVis MEDICAL SOLUTIONS | FAU FRIEDRICH-ALEXANDER UNIVERSITÄT ERLANGEN-NÜRNBERG

Thank you for taking the time to participate in our survey! Your contribution helps support our research and evaluation of the MeVis surgery planning results so that we can improve the presentation of the planning data.

Answering all questions will only take about 5 minutes. Your answers will be treated anonymously. Most of the questions are optional and can be skipped if you cannot, or do not want to, provide an answer.

If you are interested, we will be happy to share the final results of the survey with you. Information about receiving these results will be given at the end of the survey.

Please click [Start>>] to begin.

Start >>

qualtrics.com

English

Please start by sharing some demographic information.

Q2.3 **Please select your age.**

- < 30 years
- 30 - 40 years
- 40 - 50 years
- > 50 years

Q2.4 **Please select your specialization.**

- Surgery
- Radiology
- Nuclear Medicine
- Other:

Q2.5 **Please select your position <u>that matches best</u>.**

- Resident
- Attending Physician
- Consultant
- Head of Department
- Other:

<< Previous | Next >>

qualtrics.com

English

Q2.7 **For which applications do <u>you</u> use MeVis Distant Services, and how many cases do <u>you</u> work on per year?**
(Leave options blank if they do not apply.)

	Cases per year
Oncological resections	
Living donor liver transplantations	
Follow-up evaluations	
Other:	
Other:	
Other:	

Q2.8 **How many liver surgery cases take place in <u>your hospital</u> per year?**

- < 50
- 51 - 200
- 201 - 500
- > 500

<< Previous   Next >>

qualtrics.com

English

You receive the results of liver surgery planning as a 2D PDF with multiple pages, a 3D PDF with a single interactive page, and/or as a dataset for the Liver Viewer or Liver Explorer.

2D PDF

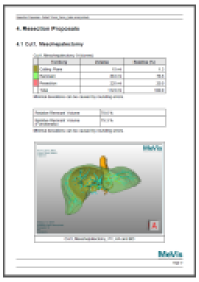

3D PDF

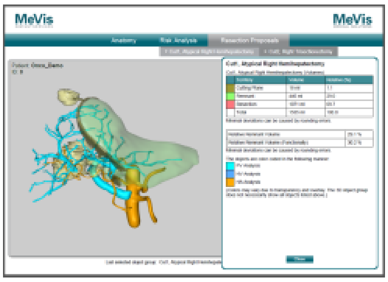

Liver Viewer / Liver Explorer

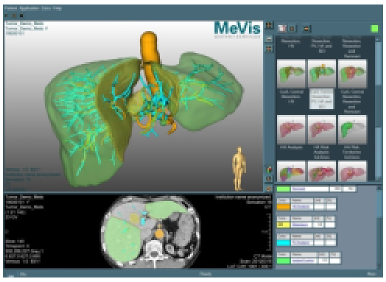

Q3.4

How often do you use each report format?

	Always	Often	Sometimes	Rarely	Never	I do not know this
2D PDF						
3D PDF						
Liver Viewer / Explorer						

<< Previous Next >>

Part 2a – for 3D PDF users only

(Participant did *not* select "Never" or "I do not know this" for question Q3.4 / Row "3D PDF").

qualtrics.com

English

Q4.2

For what purposes do you use the 3D PDF?

- [ ] Deciding whether surgery is performed
- [ ] Patient information
- [ ] Decision about surgical strategy
- [ ] Personal / mental preparation for surgery
- [ ] Support during surgery
- [ ] Tumor board discussion
- [ ] Discussion with colleagues
- [ ] Education / training
- [ ] Case presentation (e.g. conference)
- [ ] Other:

Q4.3

Do you take advantage of the possibility to exchange the 3D PDF with others?

- [ ] No
- [ ] Yes, by e-mail
- [ ] Yes, by a hardware storage medium (e.g. USB storage)
- [ ] Yes, by:

Next >>

qualtrics.com

English

Q4.5

At which workplace/device do you watch or present the 3D PDF?

- [ ] At one stationary workplace (e.g. an office PC)
- [ ] At multiple stationary workplaces
- [ ] In a conference room (e.g. using video projector or large screen)
- [ ] At a laptop / notebook
- [ ] On a tablet PC
- [ ] Other:

Q4.6

Which 3D PDF presentation options do you use in the operating room?

- [ ] None
- [ ] Paper printouts with pre-defined views
- [ ] Paper printouts with customized views
- [ ] Monitor of a PC
- [ ] Large screen (wall-mounted)
- [ ] Laptop / notebook
- [ ] Tablet PC
- [ ] Other:

<< Previous | Next >>

Q4.8

qualtrics.com

English

Please rate the statement: **The following functions and characteristics of the 3D PDFs are <u>useful</u>.**

	Agree	Somewhat agree	Somewhat disagree	Disagree	I cannot assess
Interactive zooming and viewing of the 3D model from all directions	○	○	○	○	○
Interactive selection of different views and resection proposals	○	○	○	○	○
Interactive show/hide of the liver parenchyma	○	○	○	○	○
Tables and informational text for case summary	○	○	○	○	○
Tables and informational text for the currently selected 3D view	○	○	○	○	○
Summary of all information in one page (without scrolling)	○	○	○	○	○
Grouping of all information in one file	○	○	○	○	○
Independence of pre-installed dedicated software (except Adobe Reader)	○	○	○	○	○
Other:	○	○	○	○	○
Other:	○	○	○	○	○

<< Previous   Next >>

qualtrics.com

English

Q4.10

Please rate the statement: **The following functions and properties would be useful extensions for the 3D PDF.**

	Agree	Somewhat agree	Somewhat disagree	Disagree	I cannot assess
Overlay of planning results with original radiological data	○	○	○	○	○
Individual resection planning by myself / in my hospital	○	○	○	○	○
Other:	○	○	○	○	○
Other:	○	○	○	○	○

qualtrics.com

English

Q4.12

Please rate the statement: **The 3D PDF is easy to use in the following situations.**

	Agree	Somewhat agree	Somewhat disagree	Disagree	I cannot assess
Opening / viewing	○	○	○	○	○
Download from the MDS server	○	○	○	○	○
Exchange with / forwarding to others	○	○	○	○	○
Other:	○	○	○	○	○
Other:	○	○	○	○	○

Q4.13

Would you like to use the planning data with other 3D media?

- ☐ No
- ☐ Yes, online / web viewer
- ☐ Yes, tablet viewer
- ☐ Yes, integrated into PACS
- ☐ Yes, other:

<< Previous | Next >>

Q4.15

English

Finally, rate the 3D PDF according to the contrasting word pairs below. Mark the circle closer to the word you spontaneously agree with more when you think about using the 3D PDF.

All word pairs shall have a marked choice; even if you are unsure about your agreement with any of the listed attributes or they do not apply to your experiences, please select an option.

annoying	○	○	○	○	○	○	○	enjoyable
not understandable	○	○	○	○	○	○	○	understandable
creative	○	○	○	○	○	○	○	dull
easy to learn	○	○	○	○	○	○	○	difficult to learn
fast	○	○	○	○	○	○	○	slow
inventive	○	○	○	○	○	○	○	conventional
good	○	○	○	○	○	○	○	bad
complicated	○	○	○	○	○	○	○	easy
unlikable	○	○	○	○	○	○	○	pleasing
usual	○	○	○	○	○	○	○	leading edge
unpleasant	○	○	○	○	○	○	○	pleasant
inefficient	○	○	○	○	○	○	○	efficient
clear	○	○	○	○	○	○	○	confusing
impractical	○	○	○	○	○	○	○	practical
organized	○	○	○	○	○	○	○	cluttered
attractive	○	○	○	○	○	○	○	unattractive
friendly	○	○	○	○	○	○	○	unfriendly
conservative	○	○	○	○	○	○	○	innovative

<< Previous   Next >>

Part 2b – for non 3D PDF users only
(Participant selected "Never" or "I do not know this" for question Q3.4 / Row "3D PDF").

qualtrics.com

English

Q5.2

Why do you not use the 3D PDF?

- ☐ I don't know it.
- ☐ I have not yet tried using it.
- ☐ Handling is too complicated.
- ☐ Cannot be displayed / does not work.
- ☐ I do not see any advantages over the 2D PDF.
- ☐ I do not see any advantages over the Liver Viewer / Liver Explorer.
- ☐ Other: ______

Next >>

qualtrics.com

English

Q5.4

The 3D PDF provides some functions and features not offered by the 2D PDF or the Liver Viewer.

If you were to use the 3D PDF - please rate the statement: **The following functions and characteristics of the 3D PDFs are useful.**

	Agree	Somewhat agree	Somewhat disagree	Disagree	I cannot assess
Interactive zooming and viewing of the 3D model from all directions	○	○	○	○	○
Interactive selection of different views and resection proposals	○	○	○	○	○
Interactive show/hide of the liver parenchyma	○	○	○	○	○
Tables and informational text for case summary	○	○	○	○	○
Tables and informational text for the currently selected 3D view	○	○	○	○	○
Summary of all information in one page (without scrolling)	○	○	○	○	○
Grouping of all information in one file	○	○	○	○	○
Independence of pre-installed dedicated software (except Adobe Reader)	○	○	○	○	○

<< Previous   Next >>

Part 3 – for all interviewees.

qualtrics.com

English

Thank you very much for your time and valuable answers that will help us with our aim to improve the presentation of MeVis surgery planning results.

Q6.3 Do you have further suggestions or comments?

Please click [Save Result] to finish the survey.

Save Result

qualtrics.com

Thank you, your answers have been recorded.

If you are interested in the results of this survey, please send an e-mail to mds-query@mevis.de.

Thank you very much again for your participation!

Your team of Fraunhofer MEVIS, MeVis Distant Services, and the University of Erlangen-Nuremberg.
