# Supplementary material for: Application and Evaluation of Interactive 3D PDF for Presenting and Sharing Planning Results for Liver Surgery in Clinical Routine
Source: PLoS One. 2014 Dec 31;9(12):e115697. doi: 10.1371/journal.pone.0115697 (PMC4281211; doi:10.1371/journal.pone.0115697)
Supplement: S2 File — The questionnaire (English version). PDF version of the questionnaire made of screenshots from the online survey tool. (PDF) [file pone.0115697.s002.pdf]

3D PDF report examples

The following screenshots are taken from the example 3D PDF reports provided by MeVis Medical Solutions.

These example reports are available for download:
LDLT case: http://www.mevis.de/fileadmin/files/Loesungen/demo_ldlt_english.3d.pdf
Oncological case: http://www.mevis.de/uploads/media/demo_tumor_english.3d.pdf

Please note that Adobe Reader 9.1 or later is required to properly display the reports. It is not possible to display the reports in a web browser.

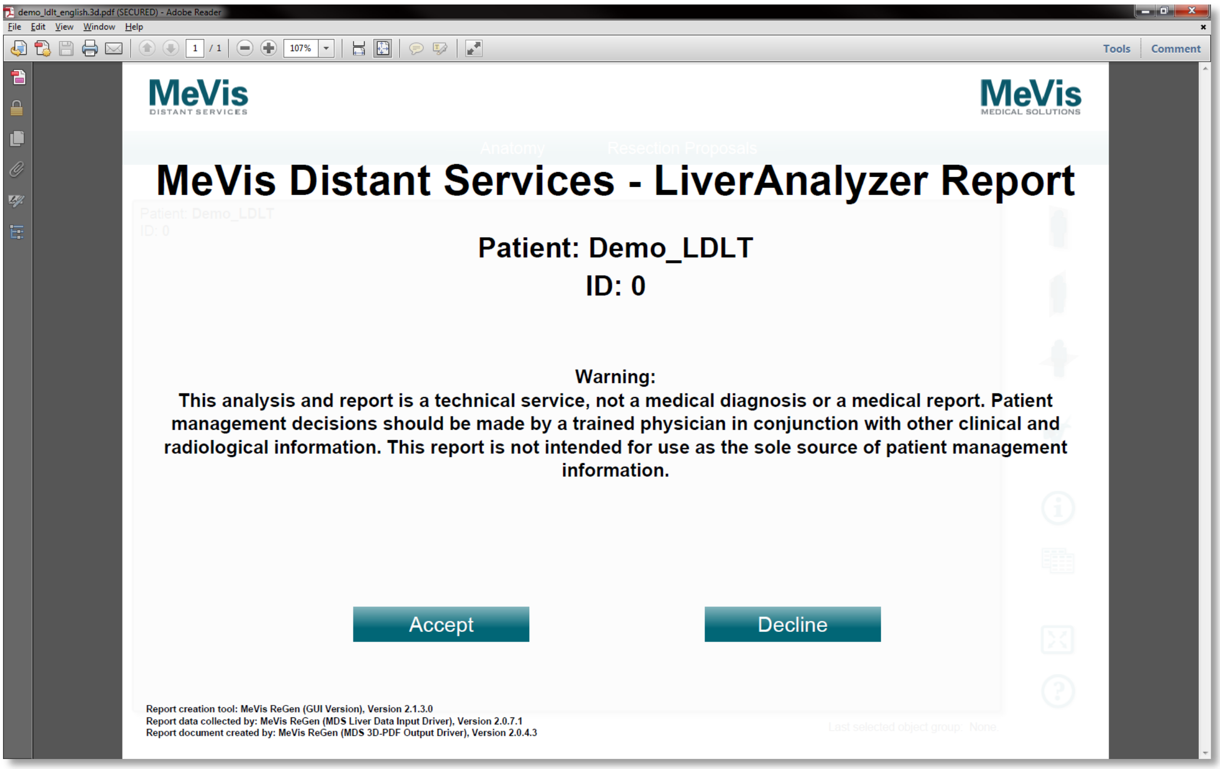

Screenshot 1: Start screen.

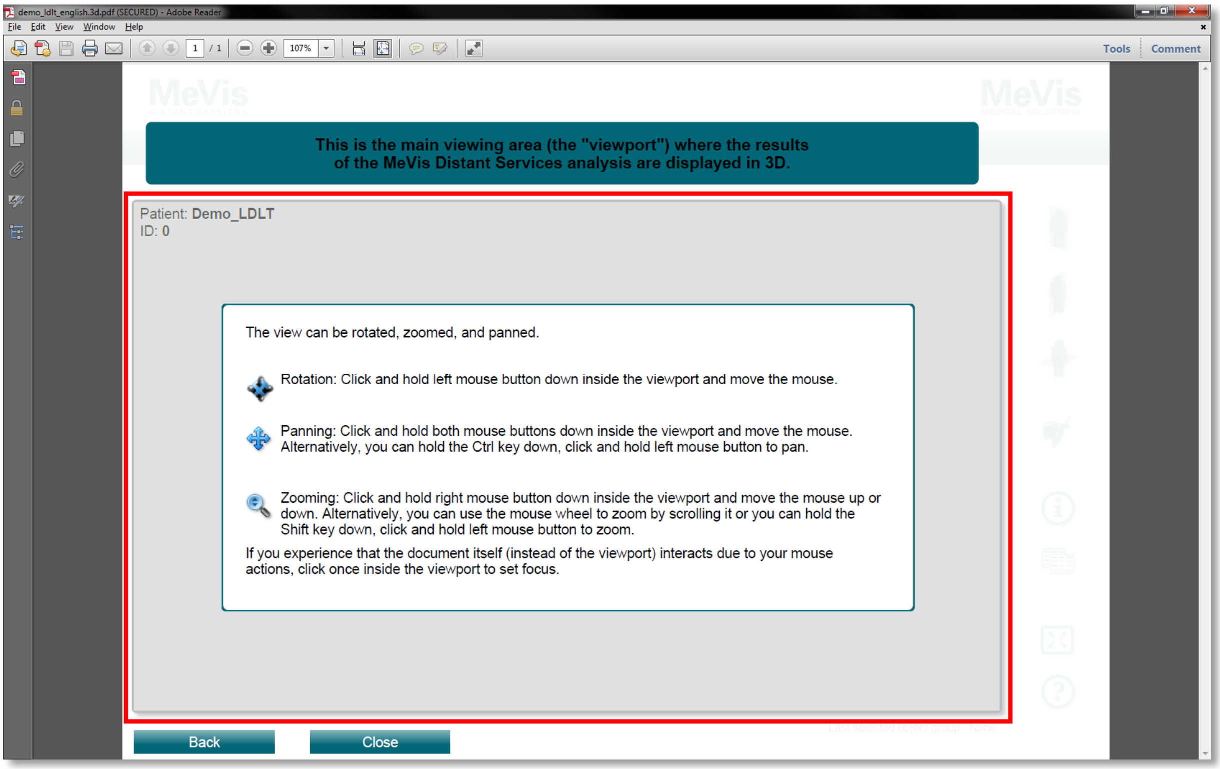

Screenshot 2: Built-in help that explains the interaction features of the 3D scene viewport.

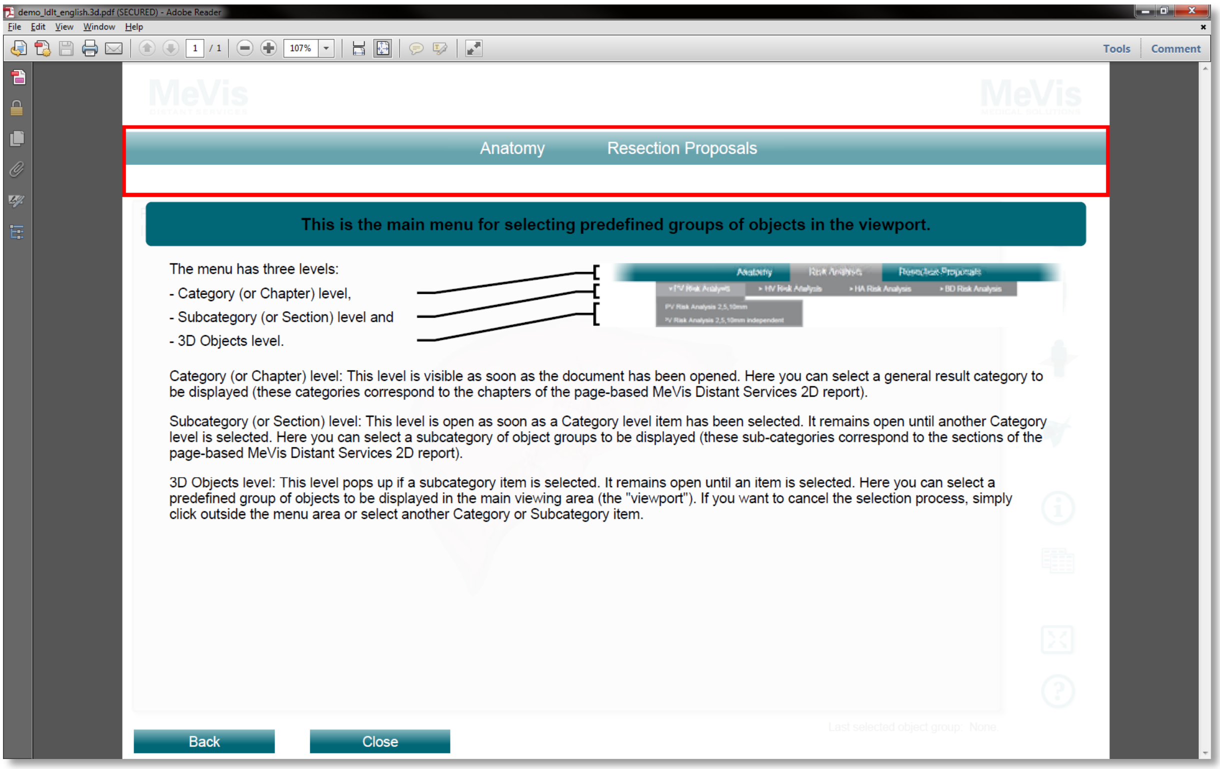

Screenshot 3: Built-in help that explains the drop-down menu.

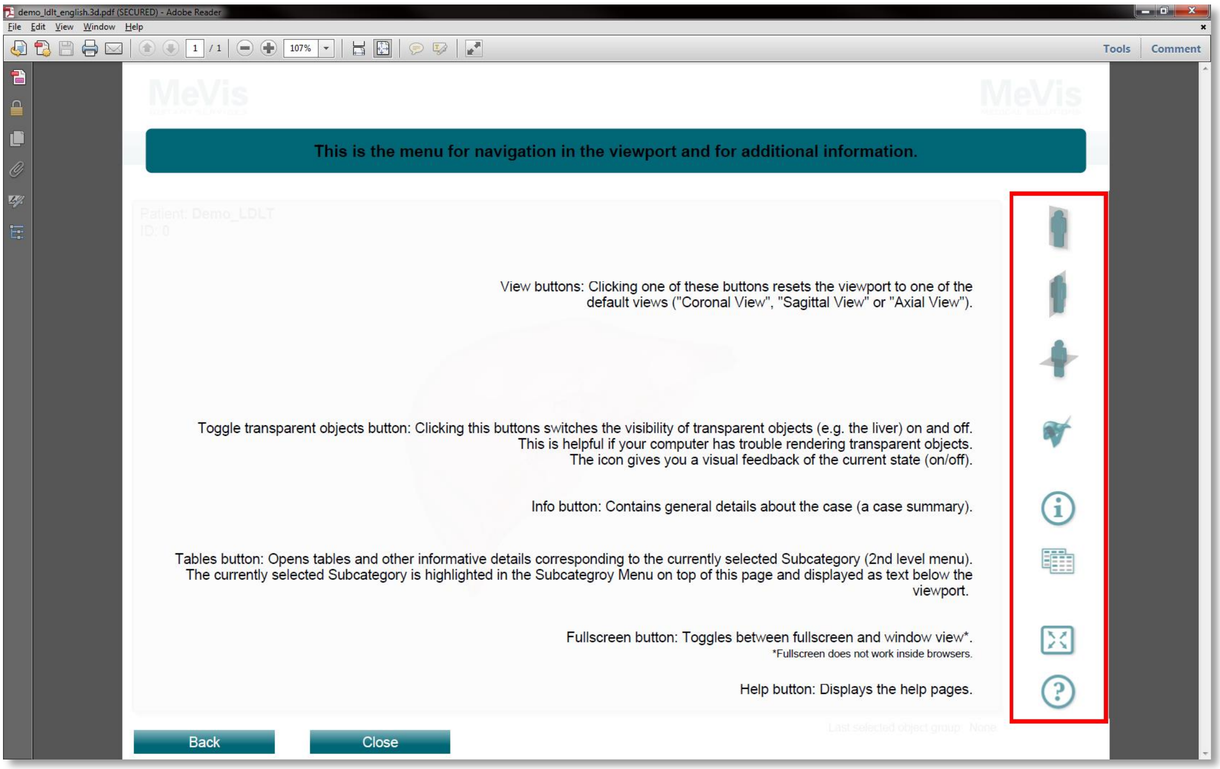

Screenshot 4: Built-in help that explains the navigation menu.

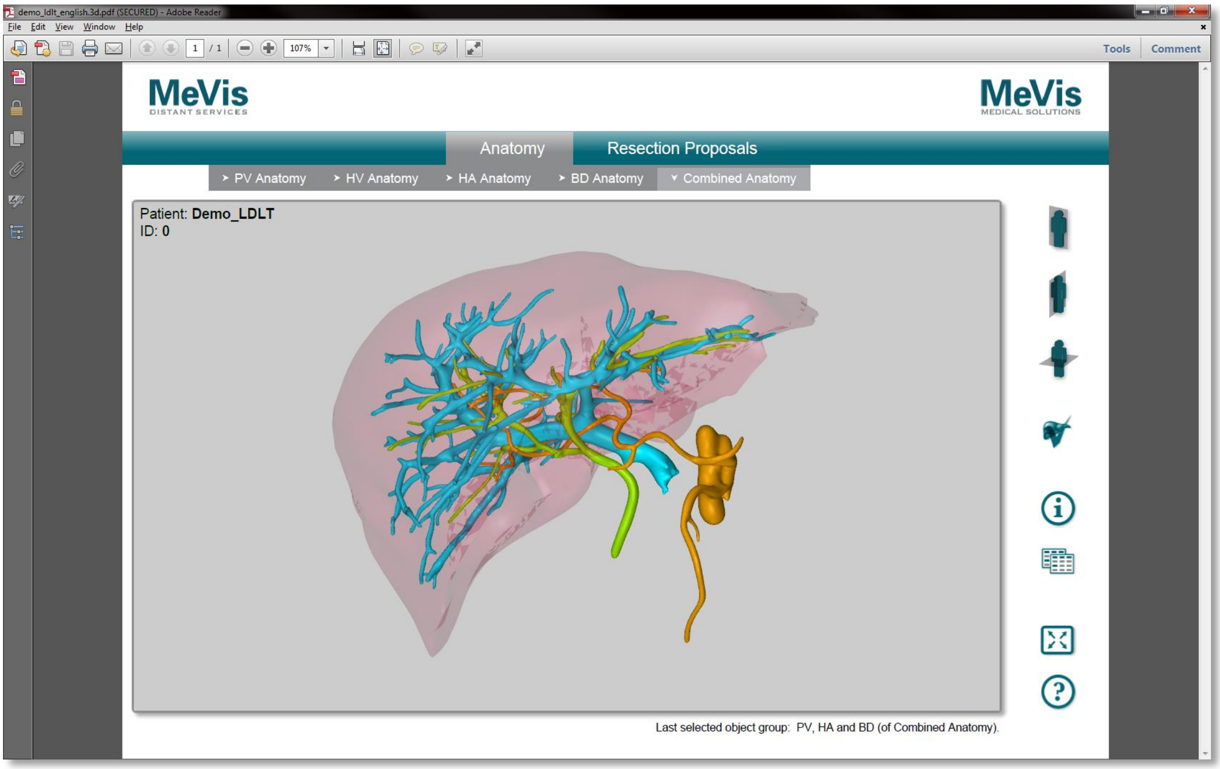

Screenshot 5: LDLT case with anatomy analysis showing portal vein, hepatic artery and bile ducts.

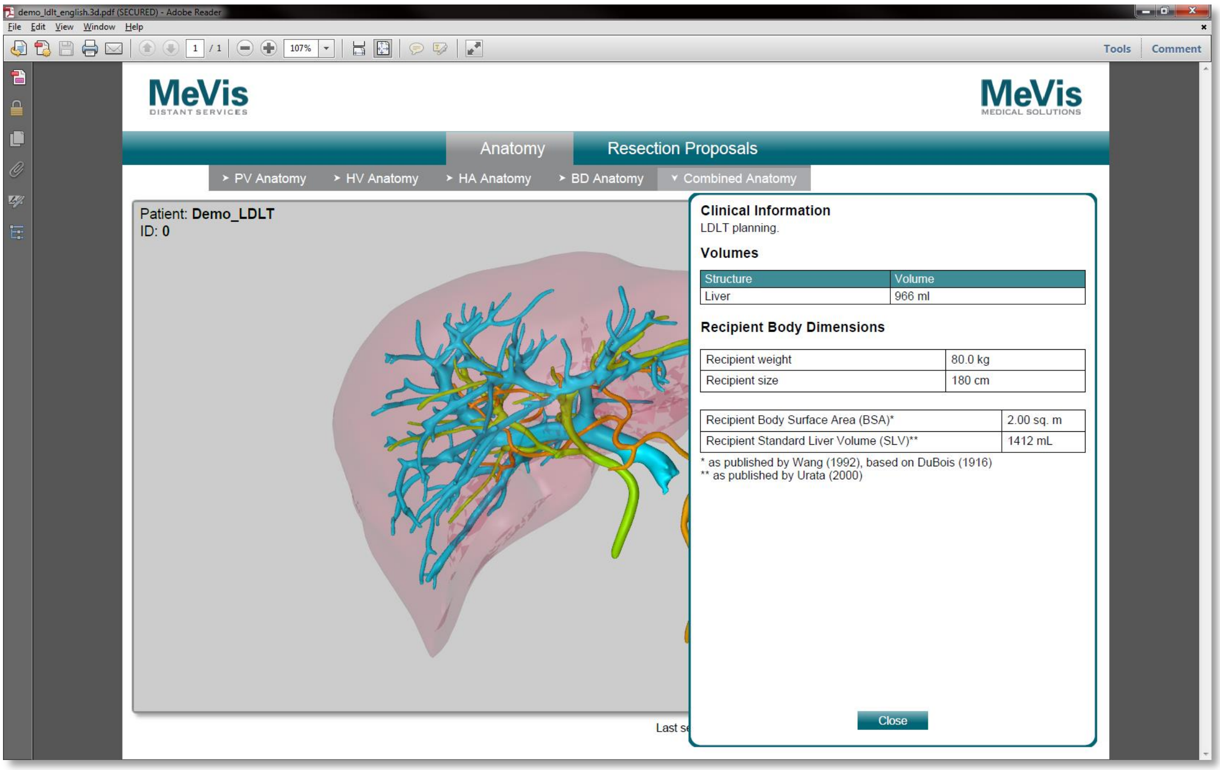

Screenshot 6: Same view as Screenshot 5, but with general case information overlay.

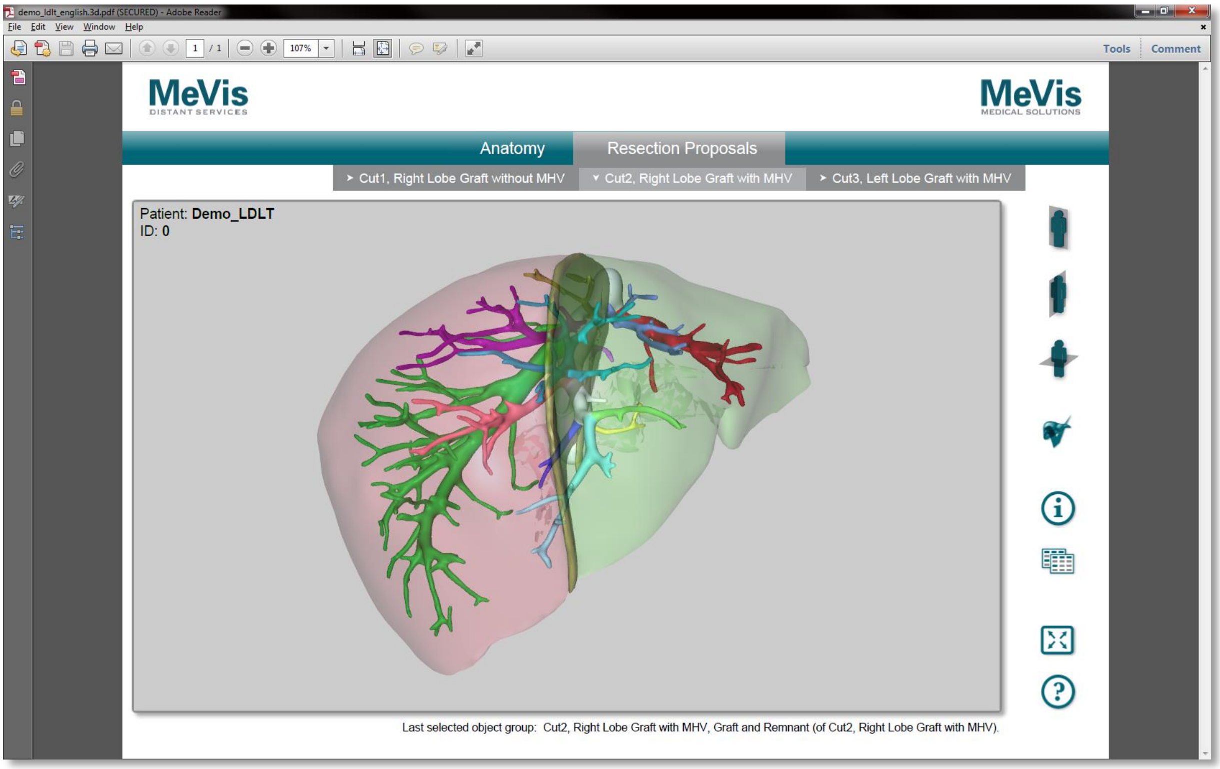

Screenshot 7: LDLT case resection proposal showing the hepatic vein (with color coded branches), the graft, the remnant and the cutting plane.

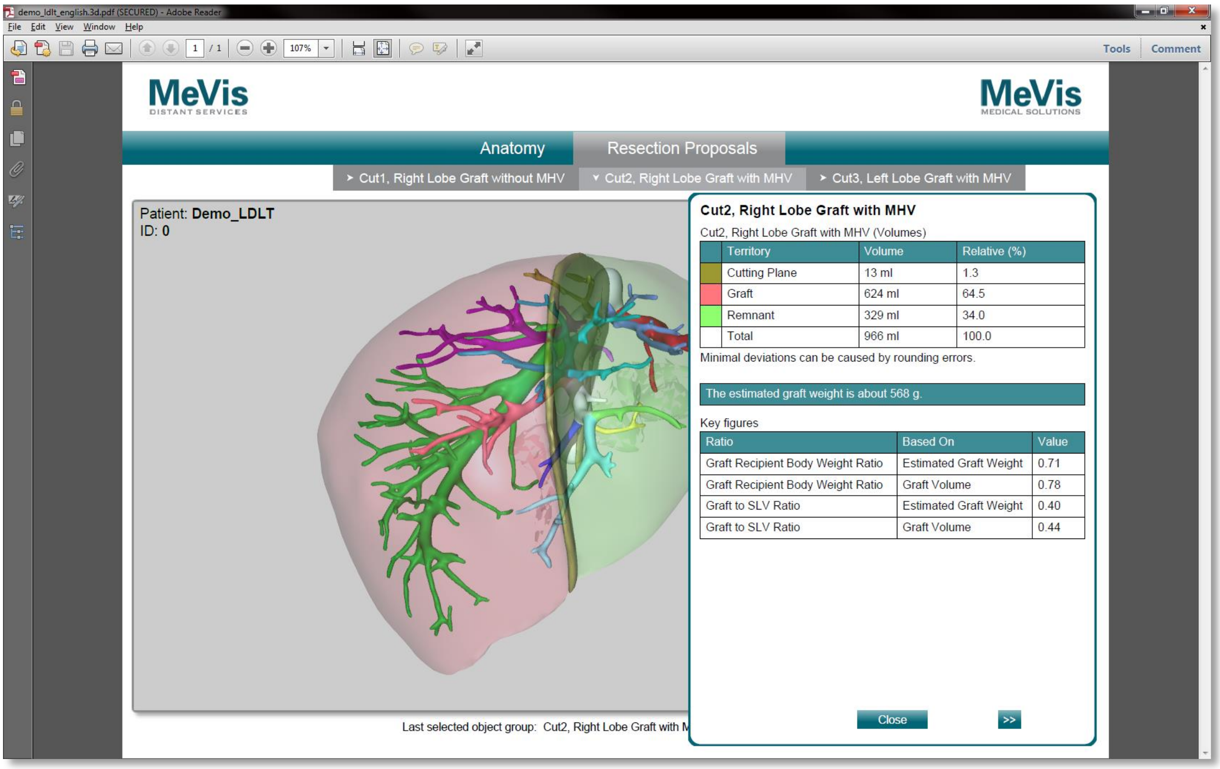

Screenshot 8: Same view as Screenshot 7, but with overlay window showing specific information for this resection proposal (first page).

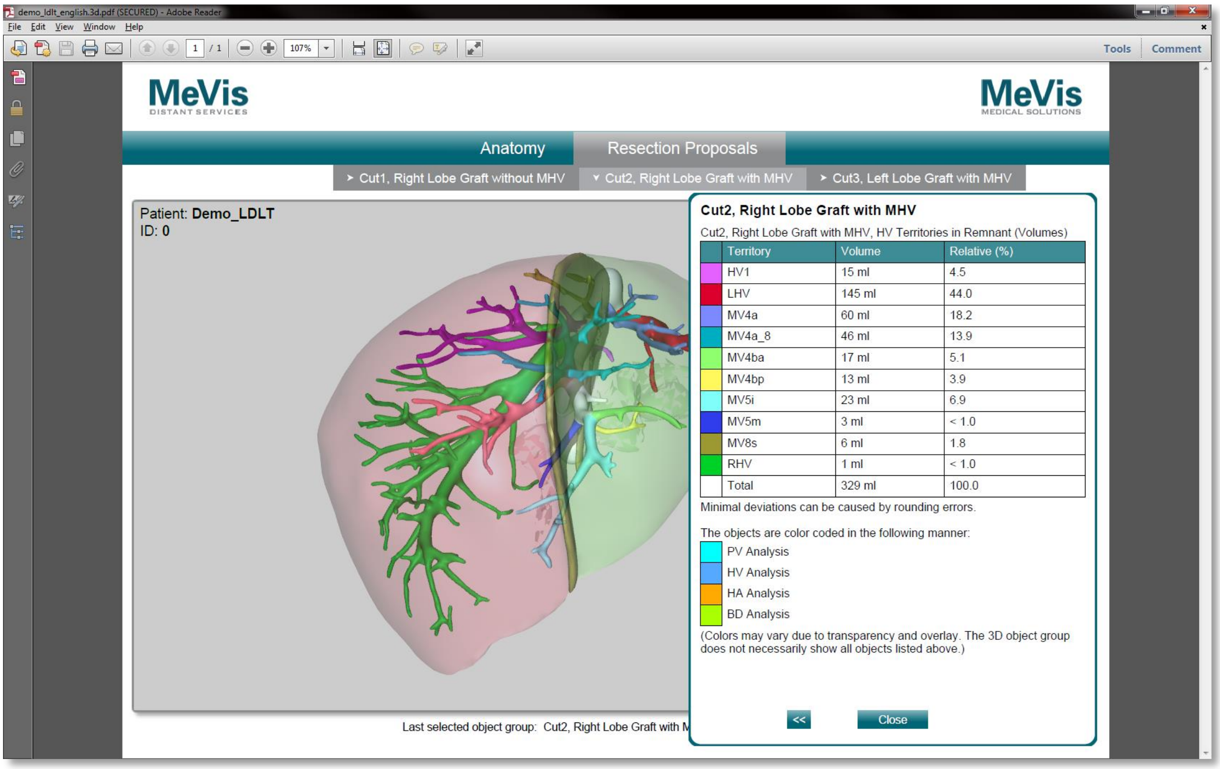

Screenshot 9: Same view as Screenshot 7, but with overlay window showing specific information for this resection proposal (second page).

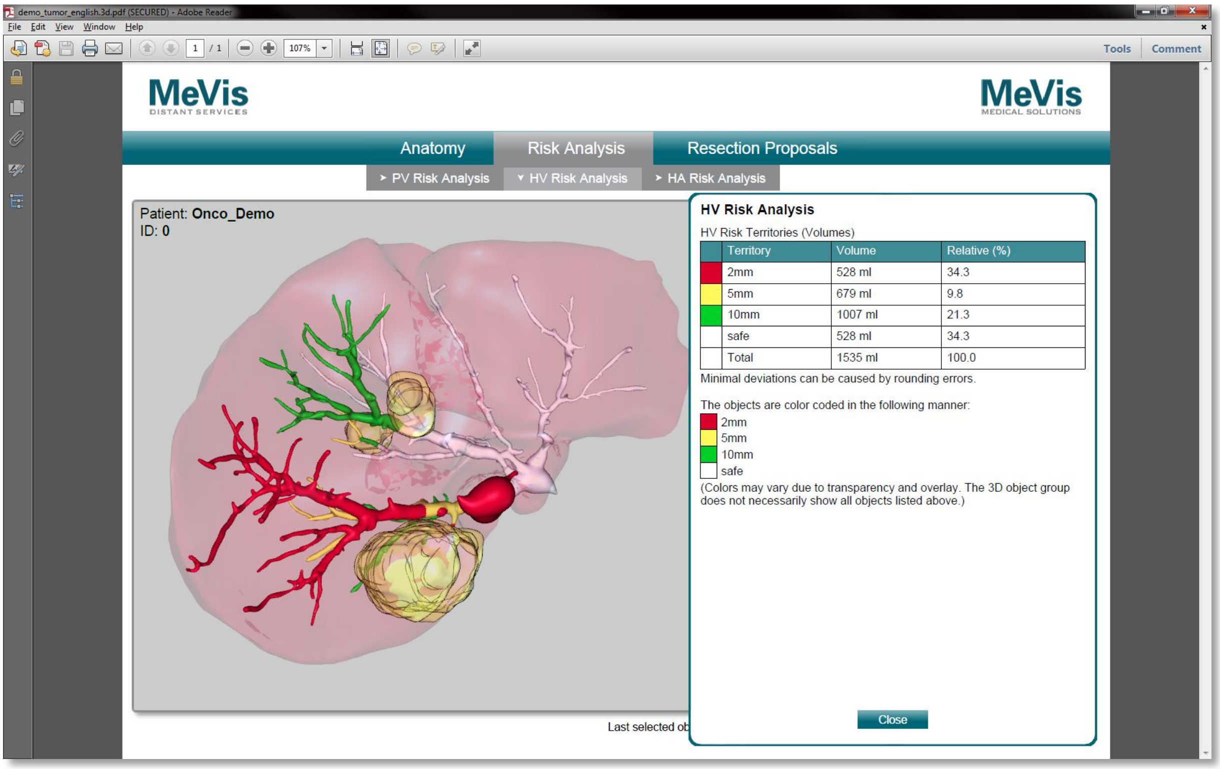

Screenshot 10: Risk analysis of a tumor case for the hepatic vein. The distance of the vessel to tumors is color coded. View from caudal to cranial.

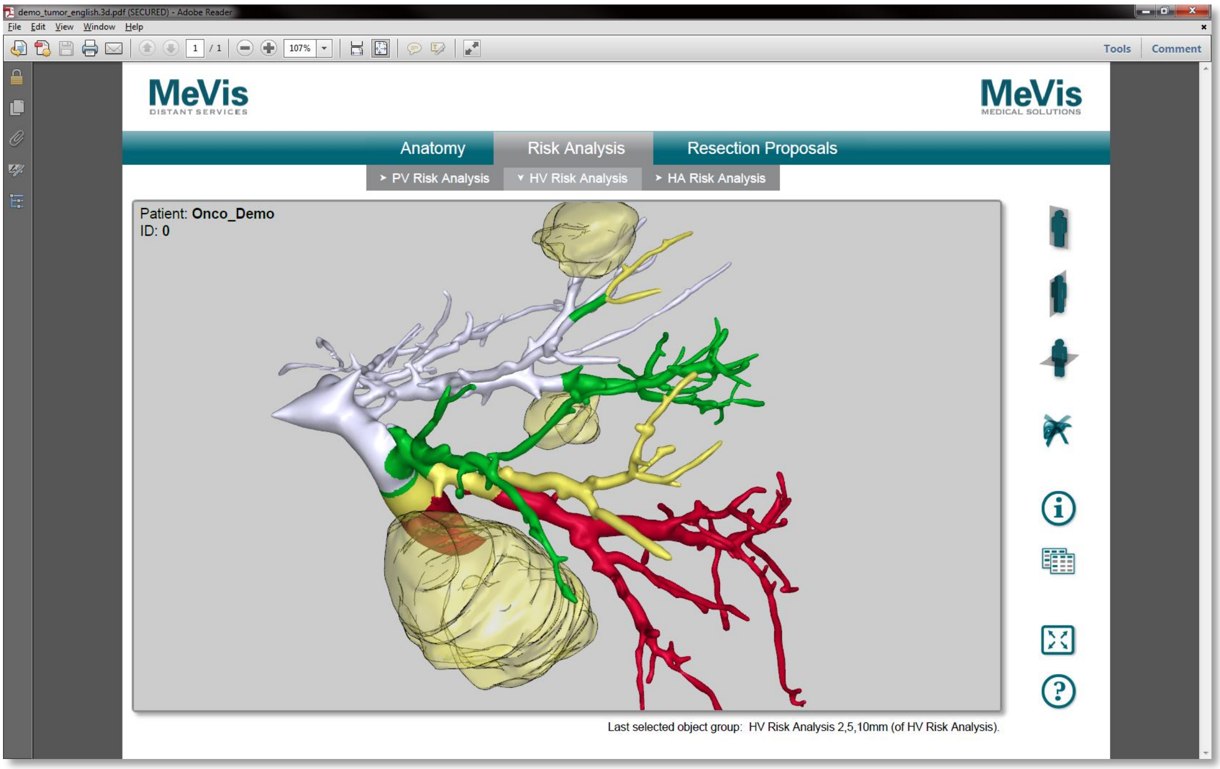

Screenshot 11: Same risk analysis as in Screenshot 10, but zoomed in and rotated to a view from cranial/dorsal.

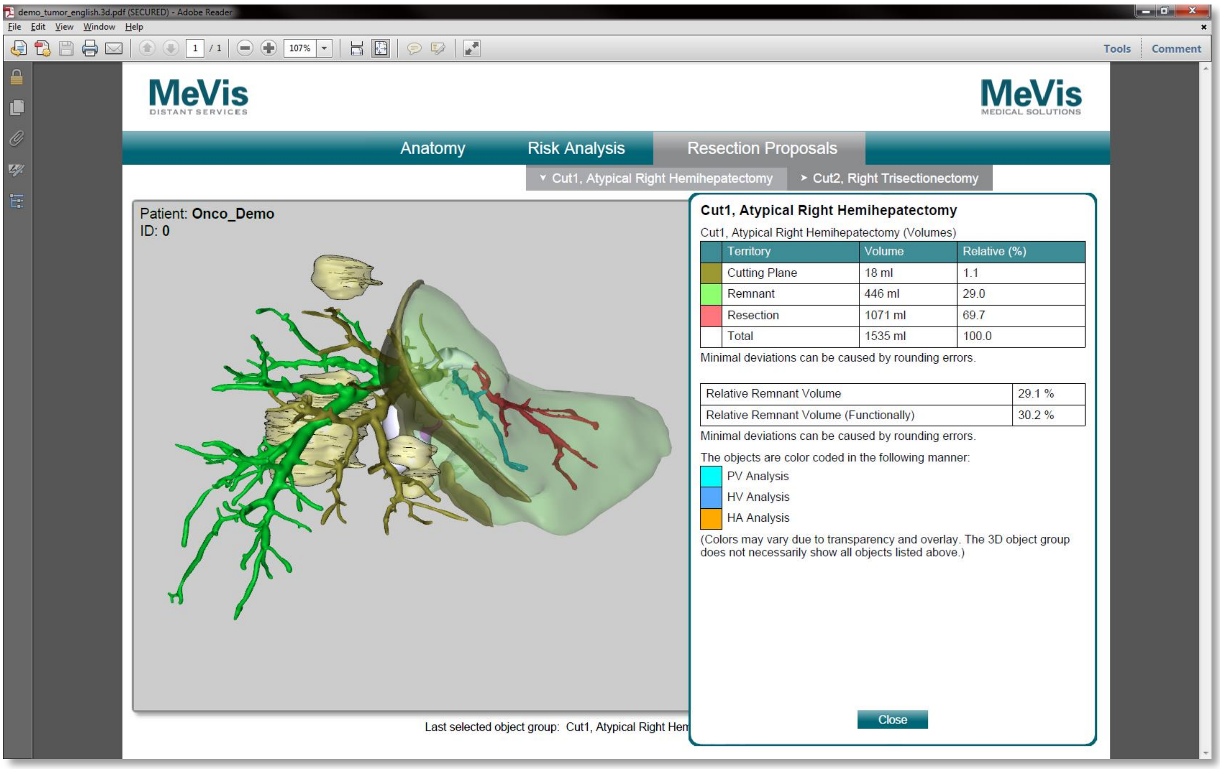

Screenshot 12: Resection proposal for a tumor case showing the hepatic vein (with color coded branches), the cutting plane, and the remnant.
